# A New Chemoenzymatic Synthesis of the Chiral Key Intermediate of the Antiepileptic Brivaracetam

**DOI:** 10.3390/molecules23092206

**Published:** 2018-08-31

**Authors:** Samuele Ciceri, Paride Grisenti, Shahrzad Reza Elahi, Patrizia Ferraboschi

**Affiliations:** 1 Department of Medical Biotechnology and Translational Medicine, Università degli Studi di Milano, Via Saldini 50, 20133 Milano, Italy; shahrzad.rezaelahi@gmail.com; 2 Chemical-Pharmaceutical Consulting and IP Management, Viale Giovanni da Cermenate 58, 20141 Milano, Italy; grisenti.paride60@gmail.com

**Keywords:** biocatalysis, lipase, irreversible transesterification, (3*R*)-3-propylbutyrolactone

## Abstract

Brivaracetam is a new anticonvulsant compound, recently approved as an antiepileptic drug. This drug substance presents a 4-substituted pyrrolidone structure: the (4*R*)-configuration of the stereocenter present on the heterocyclic ring is the main target of the synthesis. The described method allows to prepare the suitable optically pure 2-substituted primary alcohol by means of a *Pseudomonas fluorescens* lipase-catalyzed transesterification. The obtained (2*R*)-alcohol was easily transformed into the (3*R*)-3-propylbutyrolactone, an advanced intermediate of brivaracetam. The described synthetic pathway is completed with the chromatographic methods and the NMR analyses necessary to establish the chemical and the optical purity of the intermediates and of the final lactone.

## 1. Introduction

Brivaracetam (**1**, chemical name (2*S*)-2-[(4*R*)-2-oxo-4-propylpyrrolidin-1-yl]butanamide, brand name Briviact^®^) is an antiepileptic drug approved in 2016 in Europe and USA [1,2,3]. This drug substance exerts its pharmacological activity due to the high affinity for synaptic vesicle protein 2A (SV2A), which is involved in the release of chemical messengers from nerve cells [3]. This helps brivaracetam stabilize electrical activity in the brain and prevent seizures. 

**Figure 1:**
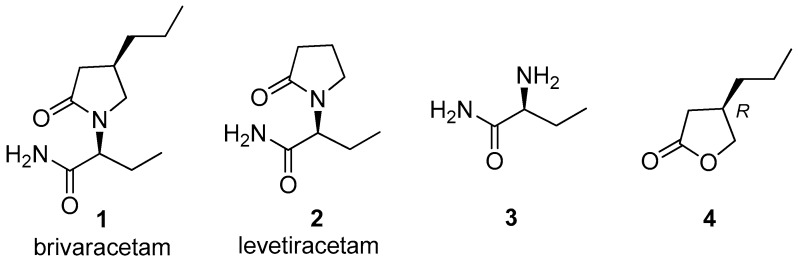
Structures of brivaracetam and related compounds.

In the structure of brivaracetam (**1**) (Figure 1) two moieties can be recognized: a pyrrolidinone ring and a butanamide; the two stereogenic centers at the 4-position of the heterocyclic ring and at the 2-position of the butanamide present an (*R*)-configuration and an (*S*)-configuration, respectively. 

The presence of the 4-propyl group provides a tenfold higher affinity for SV2A than levetiracetam (**2**), the brivaracetam analogue devoid of the 4-alkyl chain [4]. While (2*S*)-2-aminobutanamide (**3**) is a commercially available compound, the relative difficulty to find suppliers of the optically pure (4*R*)-propyl synthon stimulated us to develop a new synthetic approach to the preparation of this chiral synthon. Among the possible precursors of the *n*-propyl containing moiety we selected (3*R*)-3-propylbutyrolactone (**4**) and planned to obtain it through a biocatalytic approach. The choice to work on this chiral synthon was also motivated by the fact that this compound is a known key intermediate of the synthesis of brivaracetam. 

**Scheme 1:**
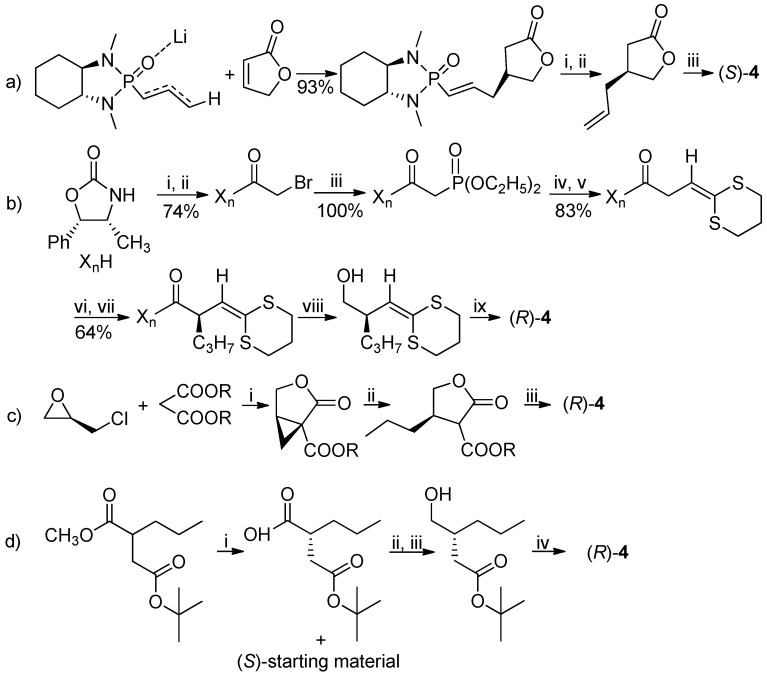
Summary of the previously reported syntheses of optically active lactone **4**. *Reagents and Conditions:* (**a**) (i) O_3_, CH_2_Cl_2_, −78 °C, (CH_3_)_2_S, 95%; (ii) CH_3_P(Ph)_3_Br, THF, nBuLi, −50 °C, 58%; (iii) H_2_, Pd/C, CHCl_3_, 99%. (**b**) (i) nBuLi, THF; (ii) ClC(O)CH_2_Br; (iii) P(OC_2_H_5_)_3_, 50 °C; (iv) NaH, THF, 25 °C; (v) 1,3-dithiane-2-carbaldehyde; (vi) LDA, −78 °C; (vii) C_3_H_7_OTf; (viii) LiAlH_4_, THF, 0 °C, 95%; (ix) TsOH, CH_2_Cl_2_, 25 °C, 90%. (**c**) (i) CH_3_ONa, C_2_H_5_OH, 55%, 98% ee; (ii) CH_3_CH_2_MgBr, CuI, 64%; (iii) LiCl, DMSO/H2O, 140 °C, 18 h, 50% overall yields of ii+iii, ee not reported. (**d**) (i) Bacillus subtilis type 2, water, 30 °C, 42% of resolved (*R*)-2-propylsuccinic acid 4-tert-butyl ester, 97.1% ee; (ii) ClCOOC_2_H_5_, (CH_3_CH_2_)_3_N, PhCH_3_, 0 °C; (iii) NaBH_4_, CH_3_OH, −20 °C; (iv) TFA, 25 °C, 91%, 95.9% ee.

The (*S*)-enantiomer of lactone **4** was already obtained in 2000 by Hanessian et al. by a phosphoramide anion addition to an α,β-unsaturated lactone followed by hydrogenation [5]. In 1993, starting from the suitably functionalized ketene dithioacetal, the (*R*)-isomer was obtained [6]. In 2016 two preparations of (*R*)-lactone **4**, utilized in the synthesis of brivaracetam, were described, starting from the (*R*)-epichlorohydrin [7] or by the enzymatic resolution of the (*R*,*S*)-methyl-2-propylsuccinate-4-*tert*-butylester [8], with >99% (of brivaracetam, after crystallization) and 95.7% ee, respectively (Scheme 1). 

**Scheme 2:**
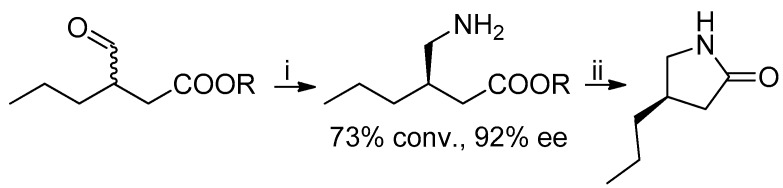
Synthesis of lactam, precursor of brivaracetam. *Reagents and Conditions*: (i) transaminase, PLP, amine donor, 30 °C, 24 h; (ii) NaOH.

In 2018, (*R*)-lactone **4** was also prepared by crystallization of the amide obtained by the reaction of (*R*,*S*)-lactone with (*S*)-1-benzylmethylamine [9]. A chemoenzymatic approach to the lactam precursor of **1**, instead of the lactone, has recently been published: in this case the suitable γ-amino-β-chiral ester was obtained by means of a transaminase-catalyzed amination with 92% ee (Scheme 2) [10].

## 2. Results and Discussion

**Scheme 3:**
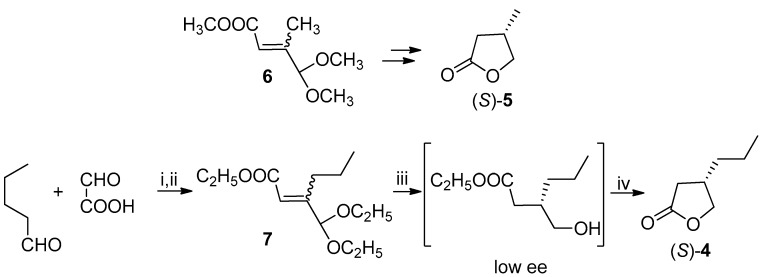
Baker’s yeast-catalyzed biotransformations. *Reagents and Conditions*: (i) See reference [15]; (ii) CH(OCH_2_CH_3_)_3_, PTSA, C_2_H_5_OH, reflux, 48 h; (iii) Baker’s yeast, 30 °C, 48 h; (iv) CF_3_COOH, rt, CH_2_Cl_2_, 10% from **7**.

Our first attempt to prepare the (*R*)-lactone **4** was carried out following the already described [11] synthesis of (3*S*)-3-methylbutyrolactone (**5**), successfully applied and studied, in the past, also by our research group [12,13,14] (Scheme 3). This method involves the stereoselective hydrogenation of the unsaturated acetal **6** by means of baker’s yeast (*Saccharomyces cerevisiae*). With this aim we prepared the suitable substrate of the microbiological transformation, compound **7**, following a reported method [15].

The presence at 3-position of a propyl chain instead of a methyl group required us to verify the stereochemical outcome of the bioreduction; indeed, the different size of the substituents at the stereogenic center, could, in principle, lead to a different chiral recognition by the microorganism.

The hydroxyl ester obtained from the biotransformation was directly cyclized [11] to known lactone **4**: the negative sign and the low optical rotation value allowed us to assign the undesired (*S*)-configuration and a low ee to the hydroxyl ester, by comparison with the reported data [5] (Scheme 3). 

Considering the very good results observed in the case of the enzymatic resolution of (2*R,S*)-2-substituted primary alcohols [16,17,18] by means of the lipase-catalyzed irreversible transesterification [19,20] we decided to prepare a (2*R*,*S*)-2-propyl primary alcohol with the C-4 engaged in an aromatic ring, a suitable precursor of the carboxylic group. The choice was addressed toward the 2-benzyl-1-pentanol (**8**), taking into account its similarity to the 2-benzyl-1-propanol (**9**), the substrate of an already reported successful resolution by means of a lipase-catalyzed irreversible transesterification [21].

The Perkin condensation between benzaldehyde and valeric anhydride afforded the (*E*)-2-benzylidenpentanoate acid (**10**) that was transformed into the corresponding ethyl ester **11** (53% yield from benzaldehyde) [22]. LiAlH_4_ in tetrahydrofuran, at reflux, afforded, by simultaneous reduction of the ester and of the double bond, the desired alcohol **8** in 69% yield.

**Scheme 4:**
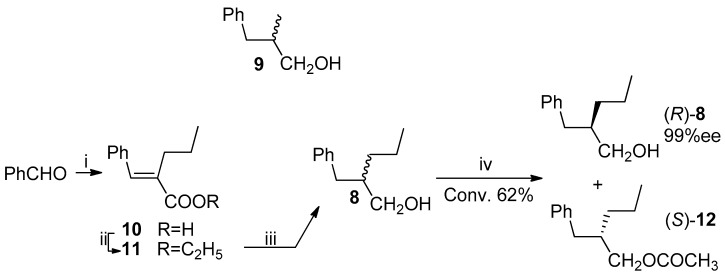
Synthesis and enzymatic resolution of alcohol **8**. *Reagents and Conditions*: (i) (C_4_H_9_CO)_2_O, CH_3_COONa, py, 140 °C, 14 h; (ii) C_2_H_5_OH, H_2_SO_4_, reflux, 12 h, 53% from PhCHO; (iii) LiAlH_4_, THF, reflux, 6 h, 69%; (iv) PFL, CH_2_=CH-OCOCH_3_, PhCH_3_, 31% of resolved (*R*)-**8** at 62% conversion.

The first attempt of resolution of the alcohol **8** was carried out under the irreversible transesterification conditions (vinyl acetate in dichloromethane) catalyzed by *Pseudomonas fluorescens* lipase (PFL), that is, under the same conditions followed in the case of the alcohol **9**. The observed (by HPLC, Hitachi Ltd., Tokyo, Japan) conversion of the (*R*,*S*)-alcohol **8** into the corresponding acetate **12** was very low, with only 35% of the ester being formed after six days. The choice to change the solvent of the enzymatic reaction from dichloromethane to toluene gave us satisfactory results: the optically pure alcohol **8** was isolated, at 62% conversion, after 48 h, a time, nevertheless, higher than the 2 h required in the case of the alcohol **9** (Scheme 4). The (*R*)-configuration of the alcohol was assigned by comparison with the reported data [23,24], and the optical purity was established by HPLC analysis on chiral stationary phase (Phenomenex Lux 3 Cellulose-1, The (*R*)- and (*S*)-acetate **12**, differently from the (*R*)- and (*S*)-alcohol **8**, were not resolved on the chiral HPLC column). The enantiomeric ratio [25,26] (E = 20), calculated for this reaction, can be regarded as moderate to good for practical purposes, according to a 2012 report of Faber and Kroutil [27].

Other common commercially available lipases did not afford satisfactory results (see Table 1). Indeed, the very fast transesterification of the (*R*,*S*)-alcohol **8** (61% conversion after 2 h) catalyzed by *Candida antarctica* lipase (CAL B, Novozym^®^ 435, Novozymes, Bagsværd, Denmark) was not stereoselective while either *Candida cylindracea* lipase (CCL) and porcine pancreas lipase (PPL) showed low enantioselectivity (about 50% ee).

Utilizing the optically pure (*R*)-alcohol **8**, obtained by the PFL-catalyzed transesterification, we proceeded with the synthesis of the (*R*)-lactone **4**.

The hydroxyl group was protected as acetate (86% yield) and the obtained ester (*R*)-**12** was submitted to the oxidative cleavage of the aromatic ring with ruthenium tetroxide, according to the method described by Sharpless et al. [28].

**Scheme 5:**
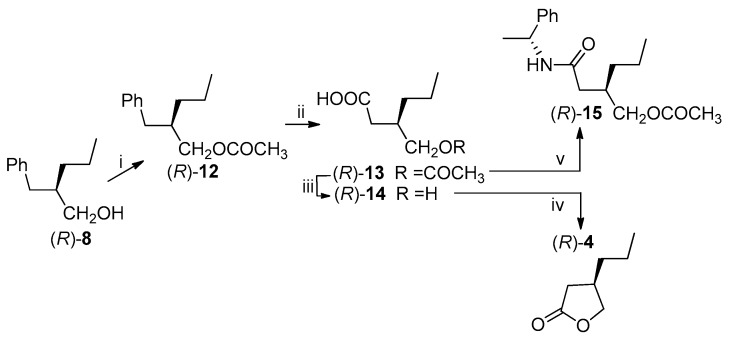
Transformation of (*R*)-alcohol **8** into (*R*)-lactone **4**. *Reagents and Conditions*: (i) (CH_3_CO)_2_O, py, rt, 2 h, 86%; (ii) RuCl_3_, NaIO_4_, CH_3_CN, ClCH_2_CH_2_Cl, H_2_O, 70 °C, 3 h, 84%; (iii) K_2_CO_3_, CH_3_OH, reflux, 3 h; (iv) 1M HCl, rt, overnight, 77% from **13**; (v) (COCl)_2_, (*R*)-(+)-α-phenylethylamine, CH_2_Cl_2_.

The acetoxy acid **13**, isolated in 84% yield, was converted into the desired (*R*)-lactone **4** by alkaline hydrolysis of the acetate followed by a direct cyclization of the obtained hydroxyacid **14** by acidic catalysis (1M hydrochloric acid). The lactone was recovered after purification in 77% yield. The ee of the intermediate **13** was established by ^1^H-NMR analysis of the diastereomeric derivative **15**, prepared by reaction with the optically pure (*R)*-phenylethylamine: the signals due to the acetyl and the CH_2_OCO- of the (3*R*)- and (3*R*,*S)*-derivatives **15** were compared confirming that the >98% ee of (*R*)-**8** was kept unaltered through the synthetic pathway. The same ee was maintained through the synthesis on the final lactone (*R*)-**4** as confirmed by chiral GC analysis (Lipodex E, Macherey-Nagel, Düren, Germany): by comparison of the chromatograms of the (*R*,*S*)-lactone, obtained from (*R*,*S*)-**8**, and of the (*R*)-lactone an ee > 98% was assigned to the (*R*)-lactone **4** (Scheme 5).

**Scheme 6:**
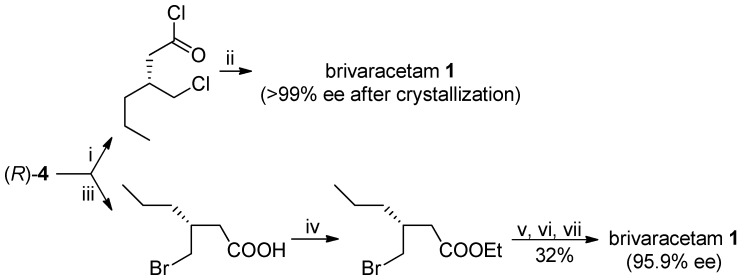
Previously reported transformations of (*R*)-lactone **4** into brivaracetam (**1**). *Reagents and Conditions:* (i) SOCl_2_, ZnCl_2_, 55 °C, 68%; (ii) (*S*)-2-aminobutanamide hydrochloride, PEG 400/CH_2_Cl_2_, −10 °C, KOH; (iii) 33% HBr in AcOH, 80 °C, 2 h, 81%; (iv) HCl, CH_3_CH_2_OH, 40 °C, 89%; (v) tetrabutylammonium iodide, (*S*)-2-aminobutanamide, Na_2_CO_3_, isopropylacetate; (vi) methyl-tert-buthyl ether; (vii) isopropylacetate.

The optically pure (*R*)-lactone **4** has been already utilized as advanced intermediate of brivaracetam: for example, the (*R*)-3-(chloromethyl)-hexanoyl chloride [7] or the ethyl ester of (*R*)-3-(bromomethyl)-3-propylbutanoic acid [8], obtained by opening of the lactone ring, were reacted with (*S*)-2-aminobutanamide to afford brivaracetam in 43% (>99% ee, after crystallization) and 23% (95.9% ee from (*R*)-lactone **4** with 95.7% ee) overall yields, respectively, with suitable specifications for its pharmaceutical use (Scheme 6).

## 3. Materials and Methods

All reagents and solvents were purchased from Sigma-Aldrich (St. Louis, MO, USA). *Candida antarctica* lipase (CAL B, Novozym^®^ 435, ≥10,000 U/g), *Candida cylindracea* lipase (CCL, 6.54 U/mg), porcine pancreas lipase (PPL, 23.9 U/mg) and *Pseudomonas fluorescens* lipase (PFL, 40.2 U/mg) were purchased from Sigma-Aldrich. 

TLC analyses were performed on silica gel 60 F_254_ precoated plates with a fluorescent indicator (Merck, Kenilworth, NJ, USA) with detection by spraying with 10% phosphomolybdic acid ethanol solution and heating at 110 °C. Column chromatographies were performed on silica gel 60 (70–230 mesh) (Merck), 1:20 *w*/*w* substrate/silica gel.

Infrared spectra of neat samples were recorded on a Perkin Elmer instrument (Mod. Paragon 500 FT-IR Spectrometer, Perkin Elmer, Waltham, MA, USA). The values of optical rotations were registered on a Perkin-Elmer (mod. 241) polarimeter in a 1 dm cell at 20 °C, setting the wavelength at 589 nm. Mass spectra were recorded on an Agilent 6330 Ion Trap instruments (ESI positive, Agilent Technologies, Santa Clara, CA, USA) using the direct inlet probe technique; the samples were dissolved in methanol at a final concentration of 0.006 mg/mL and injected at an infusion rate of 0.5 mL/h. Data acquisition and analysis were accomplished with Bruker Daltonics Data Analysis 3.3 software (Bruker Daltonics, Billerica, MA, USA). High resolution mass spectra (HRMS) were recorded on Impact HD^TM^ UHR-QqToF (Bruker Daltonics); acquisition parameters were set as following: Funnel1 RF 400 Vpp, Funnel2 RF 400 Vpp, Hexapole RF 600 Vpp, transfer time 95 µs, pre-pulse storage 12 µs, 10–1000 *m*/*z* window. Calibration was performed online using ESI-L Low Concentration Tuning Mix (Agilent Technologies); the samples were dissolved in methanol at a final concentration of 0.0006 mg/mL and injected at an infusion rate of 0.5 mL/h. Data acquisition and analysis were accomplished with Bruker Compass Data Analysis 4.1 software (Bruker Compass, Billerica, MA, USA). GC analyses were performed with a Hewlett-Packard 5890 series II (Palo Alto, CA, USA). NMR spectra were recorded on an AVANCE 500 spectrometer (Bruker, Billerica, MA, USA) equipped with a 5 mm broadband reverse probe with field *z*-gradient operating at 500.13 and 125.76 MHz for ^1^H and ^13^C, respectively. NMR spectra were recorded at 298 K in CDCl_3_ (isotopic enrichment 99.95%) solution. The data were collected and processed by XWIN-NMR software (3.5, Bruker) running on a PC with Microsoft Windows 7 (Redmond, WA, USA). The samples (10 mg), were dissolved in the appropriate solvent (0.7 mL) in a 5 mm NMR tube. Acquisition parameters for 1D were as follows: ^1^H spectral width of 5000 Hz and 32 K data points providing a digital resolution of ca. 0.305 Hz per point, relaxation delay 10 s; ^13^C spectral width of 29,412 Hz and 64 K data points providing a digital resolution of ca. 0.898 Hz per point, relaxation delay 2 s. The experimental error in the measured ^1^H-^1^H coupling constants was ±0.5 Hz. Chemical shifts (δ) of the ^1^H-NMR and ^13^C-NMR spectra are reported in ppm using the signal for residual solvent protons resonance as internal standard (^1^H-NMR: CDCl_3_ 7.26 ppm; ^13^C-NMR: CDCl_3_ 77.0 ppm (central line)). The splitting pattern abbreviations are as follows: s, singlet; d, doublet; t, triplet; q, quartet; m, multiplet, and bs, broad signal. For two-dimensional experiments, Bruker microprograms using gradient selection (gs) were applied. All two-dimensional spectra (COSY and HSQC) were acquired with 2048 data points for t_2_ and 256 for t_1_ increments. The confirmations of the structures of compounds **4**, **8**, **11**, **12**, and **13** were obtained by 500 MHz-NMR spectra, in particular through proton and carbon 1D-NMR spectra, as well as by 2D-NMR homo-correlation (COSY) and hetero-correlation (HSQC) experiments. The NMR, IR, MS and HRMS spectra and the HPLC, GC chromatograms are available in the supplementary materials section.

### 3.1. Chemistry

#### 3.1.1. (*E*)-Ethyl-2-benzylidenepentanoate (**11**)

Benzaldehyde (10 mL, 98.4 mmol), valeric anhydride (30.3 mL, 153.6 mmol), sodium acetate (4.5 g, 54.9 mmol) and pyridine (0.3 mL, 3.7 mmol) were heated at 140 °C, under stirring (14 h) monitoring the reaction progress by TLC (heptane/acetone 7:3). After cooling at room temperature, the reaction mixture was poured in an aqueous solution of hydrochloric acid (300 mL, H_2_O/37% HCl_aq_ 8:2 *v*/*v*) obtaining an emulsion. Water was removed (30 mmHg, 60 °C) to afford an oily residue. Water was added (180 mL) and the obtained aqueous phase was extracted with dichloromethane (3 × 90 mL). The collected organic phases were dried over sodium sulfate; after filtration, the solvent was removed at reduced pressure, affording desired (*E*)-2-benzylidenepentanoic acid (**10**) (14.7 g) which was used directly in the next step without any further purification. The ^1^H-NMR spectrum was in agreement with the reported one [29].

To a solution of crude (*E*)-2-benzylidenepentanoic acid (**10**, 14.7 g) in dry ethanol (180 mL), 95–97% sulfuric acid (5 mL, 93.8 mmol) was added dropwise under a nitrogen atmosphere. The solution was kept at reflux, under stirring (12 h), monitoring the reaction progress by TLC (toluene/ethyl acetate 9:1). The solution was cooled to 0–5 °C, the pH was adjusted to neutrality by the addition of a 6% aqueous ammonium hydroxide solution and ethanol was removed under reduced pressure (30 mmHg, 50 °C). The obtained aqueous phase was basified to pH 9 with 6% ammonium hydroxide solution and extracted with dichloromethane (3 × 80 mL). The collected organic phases were dried over sodium sulfate and after the usual work up a yellow oily residue (12.9 g) was obtained; the residue was purified by silica gel column chromatography by elution with hexane/ethyl acetate (95:5) giving pure (*E*)-ethyl 2-benzylidenepentanoate (**11**, 11.3 g, 51.8 mmol, 53% from benzaldehyde) as an oil. R_f_ 0.83 (toluene/ethyl acetate 9:1). ^1^H-NMR [22]: 7.65 (s, 1H, CH=), 7.30–7.41 (m, 5H, Ar), 4.27 (q, *J* = 7.1 Hz, 2H, CH_2_CH_3_), 2.49 (m, 2H, H-3), 1.53–1.62 (m, 2H, H-4), 1.35 (t, 3H, *J* = 7.1 Hz, CH_3_CH_2_), 0.96 (t, 3H, *J* = 7.3 Hz, H-5). ^13^C-NMR: 168.5 (C-1), 138.6 (PhCH=), 136.0 (Ar), 133.9 (C-2), 129.2, 128,4, 128.2 (Ar), 60.7 (OCH_2_CH_3_), 29.5 (C-3), 22.6 (C-4), 14.3 (*C*H_3_CH_2_), 14.2 (C-5). IR ν 2961.75, 2932.82, 2872.59, 1709.43, 1465.35, 1446.71, 1259.77, 1225.85, 1201.19, 1130.17, 767.24, 700.63 cm^−1^. MS (*m*/*z*) 241.5 [M + Na]^+^.

#### 3.1.2. (*R*,*S*)-2-benzylpentan-1-ol (**8**)

To a suspension of lithium aluminum hydride (9.0 g, 237.2 mmol) in dry tetrahydrofuran (150 mL), cooled at 0–5 °C, (*E*)-ethyl 2-benzylidenepentanoate (**11**) (8.5 g, 38.9 mmol) dissolved in dry tetrahydrofuran (30 mL) was added dropwise, under inert atmosphere. The ice bath was then removed, and the reaction mixture was kept at reflux (6 h); the reaction progress was monitored by TLC (toluene/ethyl acetate 9:1) until the starting material disappearance. To the reaction mixture, cooled at 0–5 °C, water (9 mL), 15% sodium hydroxide aqueous solution (9 mL), and water (27 mL) were sequentially added. The white precipitate was removed by suction through a Celite pad. The filtrate was evaporated at reduced pressure affording a light-yellow oily residue (6.9 g). The residue was purified by silica gel column chromatography by elution with hexane/ethyl acetate (9:1) to give pure (*R*,*S*)-2-benzylpentan-1-ol (**8**, 4.8 g, 26.9 mmol; 69%). R_f_ 0.40 (toluene/ethyl acetate 9:1). ^1^H-NMR [23,24]: 7.25–7.30 (m, 2H, Ar), 7.17–7.21 (m, 3H, Ar), 3.53 (d, *J* = 5.0 Hz, 2H, H-1), 2.64 (d, *J* = 7.3, 2H, PhCH_2_,), 1.81 (m, 1H, H-2), 1.24–1.45 (m, 5H, H-3, H-4 and exchangeable with D_2_O), 0.90 (t, *J* = 7.5 Hz, 3H, H-5). ^13^C-NMR [23,24]: 140.8, 129.2, 128.3, 125.8 (Ar), 64.9 (C-1), 42.4 (C-2), 37.7 (PhCH_2_), 33.1 (C-3), 20.1 (C-4), 14.3 (*C*-5). IR [23,24] ν 3343.95, 3084.85, 3062.51, 3026.81, 2957.07, 2928.70, 1603.22, 1495.59, 1465.77, 1454.37, 1040.02, 732.92, 699.94 cm^−1^. MS (*m*/*z*) 201.0 [M + Na]^+^.

#### 3.1.3. (*R*)-2-benzylpentan-1-ol (**8**)

To a solution of (*R*,*S*)-2-benzylpentan-1-ol (**8**, 1.8 g, 10.2 mmol) in toluene (900 mL), vinyl acetate (2.82 mL, 31 mmol) and PFL (180 mg, 40.2 U/mg) were sequentially added. The reaction mixture was kept at 20 °C under vigorous stirring in a screw cap flask. The reaction progress and the ee of alcohol **8** were monitored by HPLC (column: Phenomenex Lux 3 Cellulose-1, 250 × 4.6 mm; UV detector wavelength 220 nm, eluant: *n*-hexane/2-propanol 98:2; flow rate 0.5 mL × min^−1^). Rt (*R*)-alcohol **8** 43.74 min; acetate **12** 9.40 min (see also footnote in the text). The reaction was stopped when alcohol **8** showed 99% ee (62% conversion); the enzyme was removed by filtration and the solvent was evaporated at reduced pressure. The oily residue (1.8 g) was purified by silica gel column chromatography. By elution with hexane/ethyl acetate 95:5, acetate (*S*)-**12** was recovered (1.28 g, 57%). Elution with hexane/ethyl acetate 9:1 afforded alcohol (*R*)-**8** (0.56 g, 31%). [α]${}_{D}^{20}$ + 9.4 (*c* 1, methanol). For the (*S*)-enantiomer lit. [23] −9.9 (*c* 1.09, methanol).

#### 3.1.4. (*R*)-2-benzylpentan-1-ol, Acetate (**12**)

To a solution of (*R*)-2-benzylpentan-1-ol (**8**, 0.440 g, 2.49 mmol) in dry pyridine (3.75 mL), acetic anhydride (0.75 mL, 7.8 mmol) was added under inert atmosphere. The reaction mixture was kept at room temperature under stirring for 2 h. The reaction progress was monitored by TLC (toluene/ethyl acetate 9:1) until starting material disappearance. Water (6 mL) was added and the mixture was extracted with dichloromethane (3 × 7.5 mL). The collected organic phases were washed with water (3 × 6 mL), dried over sodium sulfate and filtered. Removal of the solvent at reduced pressure gave title compound (*R*)-2-benzylpentyl acetate (**12**) as a light-yellow oil (0.470 mg, 2.13 mmol, 86%) which was used in the next step without further purification. For analytical purposes a little amount (16 mg) was purified by silica gel column chromatography. Elution with hexane/ethyl acetate 95:5 afforded pure acetate-**12**. R_f_ 0.76 (toluene/ethyl acetate 9:1). ^1^H-NMR: 7.25–7.31 (m, 2H, Ar), 7.13–7.21 (m, 3H, Ar), 3.95 (d, *J* = 5.3 Hz, 2H, H-1), 2.63 (d, *J* = 7.0 Hz, 2H, PhCH_2_), 2.04 (s, 3H, CH_3_), 1.98 (m, 1H, H-2), 1.26–1.40 (m, 4H, H-3 and H-4), 0.93 (t, *J* = 7.0 Hz, 3H, H-5). ^13^C-NMR: 171.2 (CO), 140.2, 129.1, 128.3, 125.9 (Ar), 66.5 (C-1), 39.1 (C-2), 37.9 (Ph*C*H_2_), 33.3 (C-3), 20.9 (*C*H_3_CO), 19.9 (C-4), 14.2 (C-5). IR ν 3085.86, 3063.45, 3027.82, 2958.22, 2872.73, 1738.63, 1603.76, 1495.90, 1466.58, 1455.21, 1385.12, 1365.87, 1239.08, 1039.32, 734.54, 700.64 cm^−1^. [α]${}_{D}^{20}$ − 14.1 (*c* 1, chloroform). MS (*m*/*z*) 243.0 [M + Na]^+^. HRMS (ESI) *m*/*z*: Calcd. for C_14_H_20_NaO_2_ [M + Na]^+^ 243.1361. Found 243.1354.

#### 3.1.5. (*R*)-3-(acetoxymethyl)hexanoic Acid (**13**)

To a mixture of (*R)*-2-benzylpentyl acetate (**12**, 0.42 g, 1.92 mmol), acetonitrile (7.5 mL), 1,2-dichloroethane (7.5 mL) and water (19 mL), ruthenium (III) chloride (77 mg, 0.36 mmol) and sodium periodate (8.7 g, 40.8 mmol) were sequentially added. The reaction mixture was kept at 70 °C under stirring (3 h), monitoring the reaction progress by TLC (hexane/acetone 8:2). The reaction mixture was cooled at room temperature and filtered through a Celite pad, washing the pad with dichloromethane (15 mL). The organic phase was separated, and the aqueous phase was extracted with dichloromethane (2 × 15 mL). The collected organic phases were dried over sodium sulfate, filtered and concentrated under reduced pressure to afford a dark violet oil. The oil was taken up in diethyl ether and passed through a pad of Celite and Florisil (1:1). The solvent was removed under reduced pressure affording (*R*)-3-(acetoxymethyl)hexanoic acid **13** (300 mg, 1.59 mmol, 84%) which was used in the next step without further purification. For analytical purposes a sample (40 mg) was purified by silica gel column chromatography. Elution with hexane/acetone 85:15 afforded pure title compound **13**. R_f_ 0.39 (hexane/acetone 8:2). ^1^H-NMR: 4.12 (dd, *J* = 4.8 and 10.8 Hz, 1H, CH_a_–OCO), 3.95 (dd, *J* = 6.8 and 10.8 Hz, CH_b_–OCO), 2.38 (m, 2H, H-2), 2.23 (m, 1H, H-3), 2.05 (s, 3H, CH_3_CO), 1.30–1.40 (m, 4H, H-4 and H-5), 0.91 (t, *J* = 6.9 Hz, 3H, H-6). ^13^C-NMR: 178.8 (C-1), 171.1 (CH_3_CO), 66.5 (CH_2_O), 36.5 (C-2), 34.3 (C-3), 33.3 (C-4), 20.8 (CH_3_CO), 19.8 (C-5), 14.1 (C-6). IR ν 3450.00, 3105.26, 2960.66, 2874.87, 2673.68, 1741.03, 1708.51, 1242.13, 1041.89 cm^−1^. [α]${}_{D}^{20}$ + 3.5 (*c* 1, chloroform). MS (*m*/*z*) 210.9 [M + Na]^+^. HRMS (ESI) *m*/*z*: Calcd. for C_9_H_15_Na_2_O_4_ [M-H + 2Na]^+^ 233.0766. Found 233.0761.

#### 3.1.6. (*R*)-4-propyldihydrofuran-2(3H)-one (**4**)

To a solution of (*R*)-3-(acetoxymethyl)hexanoic acid (**13**, 145 mg, 0.78 mmol) in methanol (13 mL) potassium carbonate (0.600 g, 4.35 mmol) was added. The reaction mixture was kept at reflux under stirring (3 h), monitoring the reaction progress by TLC (hexane/acetone 8:2) until starting material disappearance. The solvent was removed at reduced pressure (30 mmHg, 40 °C) and the residue was taken up with water (6 mL). The solution of hydroxyacid **14** was cooled at 0–5 °C and pH was adjusted to 3 by addition of a 1M aqueous hydrogen chloride solution. The solution was stirred at room temperature overnight. The reaction progress was monitored by TLC (hexane/acetone 8:2). The reaction mixture was extracted with dichloromethane (3 × 15 mL). The collected organic phases were dried over sodium sulfate; after filtration, the solvent was removed at reduced pressure, to afford crude (*R*)-4-propyldihydrofuran-2(3*H*)-one (**4**, 102 mg) which was purified by silica gel column chromatography. Elution with hexane/acetone 9:1 afforded pure (*R*)-**4** (75 mg, 0.60 mmol, 77%). R_f_ 0.55 (hexane/acetone 8:2). GC (column: Macherey-Nagel Lipodex E, 25m × 250 μm; FID detector at 220 °C, carrier gas helium; injector 200 °C; column inlet pressure 60 kPa; split ratio 1:100 gradient: 68 min run (70 °C/1 min, 70 to 122 °C/52 min, 122 °C/15 min). Rt (*R*)-lactone **4** 57.75 min. The (*S*)-lactone peak (Rt 58.57 min) is not detectable. ^1^H-NMR [5,6]: 4.41 (dd, *J* = 7.3 and 9.2 Hz, H_a_-5), 3.92 (dd, *J* = 7.1 and 9.3 Hz, H_b_-5), 2.50–2.65 (m, 2H, H_a_-3 and H-4), 2.17 (dd, *J*= 7.8 and 16.1 Hz, H_b_-3), 1.42–1.49 (m, 2H, CH_2_–CH), 1.29–1.39 (m, 2H, CH_2_CH_3_), 0.93 (t, *J* = 7.3 Hz, 3H, CH_3_). ^13^C-NMR [5,6]: 177.2 (C-2), 73.4 (C-5), 35.5 (C-4), 35.3 (CH_2_–CH), 34.5 (C-3), 20.6 (CH_2_CH_3_), 13.9 (CH_3_). IR [6] ν 3530.81, 2960.55, 2930.02, 2873.93, 1774.46, 1466.22, 1420.21, 1379.61, 1170.61, 1015.84 cm^−1^. [α]${}_{D}^{20}$ + 8.4 (*c* 1, ethanol). For for (*S*)-enantiomer lit. [5] −5.6 (*c* 0.9, ethanol), for (*R*)-enantiomer lit. [6] +6.7 (*c* 3.9, ethanol). MS (*m*/*z*) 150.9 [M + Na]^+^.

#### 3.1.7. Diastereomeric Derivative **15** of (R)-3-(acetoxymethyl)hexanoic Acid (**13**)

A solution of the acetoxy acid **13** (18.7 mg, 0.1 mmol) in anhydrous dichloromethane (0.5 mL) was treated with oxalyl chloride (11 μL, 0.13 mmol), in the presence of dimethylformamide (20 μL), at 0 °C. After 1 h, the solution was concentrated under nitrogen flow and (*R*)-(+)-methylbenzylamine (52 μL, 0.4 mmol) was added. After 30’ ethyl acetate (2 mL) was added. The obtained solution was sequentially washed with 6M hydrochloric acid (3 × 1 mL), 3% aqueous ammonium hydroxide solution (3 × 1 mL), water (2 mL); finally, solvent was removed at reduced pressure.

For the 500 MHz ^1^H-NMR analysis, the resonances of the (*R*)- and (*S*)-CH_2_OCOCH_3_ or the CH_3_CO group were first established from spectra of the derivative of (*R*,*S*)-**13**, then they were compared with that of the derivative of (*R*)-3-(acetoxymethyl)hexanoic acid **13**. In the spectrum of derivative of (*R*,*S*)-**13** two singlets at 1.94 and 2.05 ppm were present, due to the CH_3_CO group protons. In the spectrum of derivative of (*R*)-**13** only the singlet at 1.94 ppm was present. The signal due to the proton H_a_ of CH_2_OCO group was a double doublet centered at 4.01 ppm for the derivative of (*R*)-**13**, while two dd (centered at 4.05 and 4.01 ppm) were present in the spectrum of amide of (*R*,*S*)-**13**. 

## 4. Conclusions

The optically pure key intermediate (*R*)-lactone **4** of brivaracetam (**1**), an orally or intravenously administered antiepileptic drug, was prepared by means of an enzymatic resolution, under the classical irreversible transesterifications conditions, of a suitable alcohol easily prepared in 36% overall yields starting from benzaldehyde. The transformation of the (*R*)-alcohol **8** into the final (*R*)-lactone **4** was efficiently accomplished in few steps, keeping unaltered the ee of the starting material. The whole synthesis involves simple steps and it can also be employed for preparative purposes; ruthenium tetroxide is used in catalytic amount due to its continue recycling by sodium periodate. Only few chromatographic purifications are required, appreciable amounts of by-products being formed only during the Perkin condensation. The relatively low yield (53%) of this step, however, can be considered acceptable taking into consideration the cheap starting materials utilized, benzaldehyde and valeric anhydride. 

The PFL-catalyzed acetylation applied to the resolution of 2-substituted primary alcohols, under the irreversible transesterification conditions, once again, gave excellent results, showing the enantiopreference in agreement with the Kazlauskas rule [30]: the modified reaction conditions (i.e., different solvent, increased enzymatic units/substrate mmoles ratio, and longer reaction time) respect to the resolution of the very similar alcohol **9** are, probably, due to a different preparation of commercial PFL and to the larger hindrance of the 2-substituent of the enzymatic substrate. The optimization of the analytical chromatographic methods (GC and HPLC) on chiral stationary phases, aimed to the evaluation of the ee of the chiral compounds, and the careful NMR analyses provide a useful complement of the synthesis.

## Figures and Tables

**Table 1:** Enzymatic irreversible transesterification of compound **8.**

Entry	Enzyme	Solvent	Time (h)	Conversion (%) ^a^	Ee ^a^, Config 8	E
1	PFL	Toluene	48	62	99, *R*	20
2	PFL	Dichloromethane	144	35	81 ^b^, *S*	15
3	CCL	Toluene	24	72	58, *S*	3
4	CAL B	Toluene	2	61	5, *R*	1
5	PPL	Toluene	192	71	53, *S*	2

^a^ by HPLC analysis on chiral stationary phase. ^b^ in this case the ee of the (*S*)-alcohol, obtained by hydrolysis (5% NaOH in CH_3_OH) of the (*S)*-acetate **12**, was evaluated.

## Supplementary Materials

The NMR, IR, MS and HRMS spectra and the HPLC, GC chromatograms are available online.
