# Supplementary material for: A New Chemoenzymatic Synthesis of the Chiral Key Intermediate of the Antiepileptic Brivaracetam
Source: Molecules. 2018 Aug 31;23(9):2206. doi: 10.3390/molecules23092206 (PMC6225152; doi:10.3390/molecules23092206)

Supporting information

A new chemoenzymatic synthesis of the chiral key intermediate of the antiepileptic brivaracetam

Samuele Ciceri,[a] Paride Grisenti,[b] Shahrzad Reza Elahi,[a] and Patrizia Ferraboschi.[a,*]

[a]Dipartimento di Biotecnologie Mediche e Medicina Traslazionale, Università degli Studi di Milano, Via Saldini 50, 20133 Milano, Italy;
shahrzad.rezaelahi@gmail.com (S.R.)
[b]Chemical-Pharmaceutical Consulting and IP Management, Viale Giovanni da Cermenate 58, 20141 Milano, Italy;
grisenti.paride60@gmail.com (P.G.)
* Correspondence: patrizia.ferraboschi@unimi.it (P.F.); samuele.ciceri@guest.unimi.it (S.C.)
Tel. +039-02-50316052

Table of contents

NMR Spectra	Page S-2
IR	Page S-9
MS	Page S-14
HRMS	Page S-19
HPLC analysis	Page S-20
GC analysis	Page S-21

^{1}H and ^{13}C NMR spectra

(E)-Ethyl-2-benzylidenepentanoate **11**

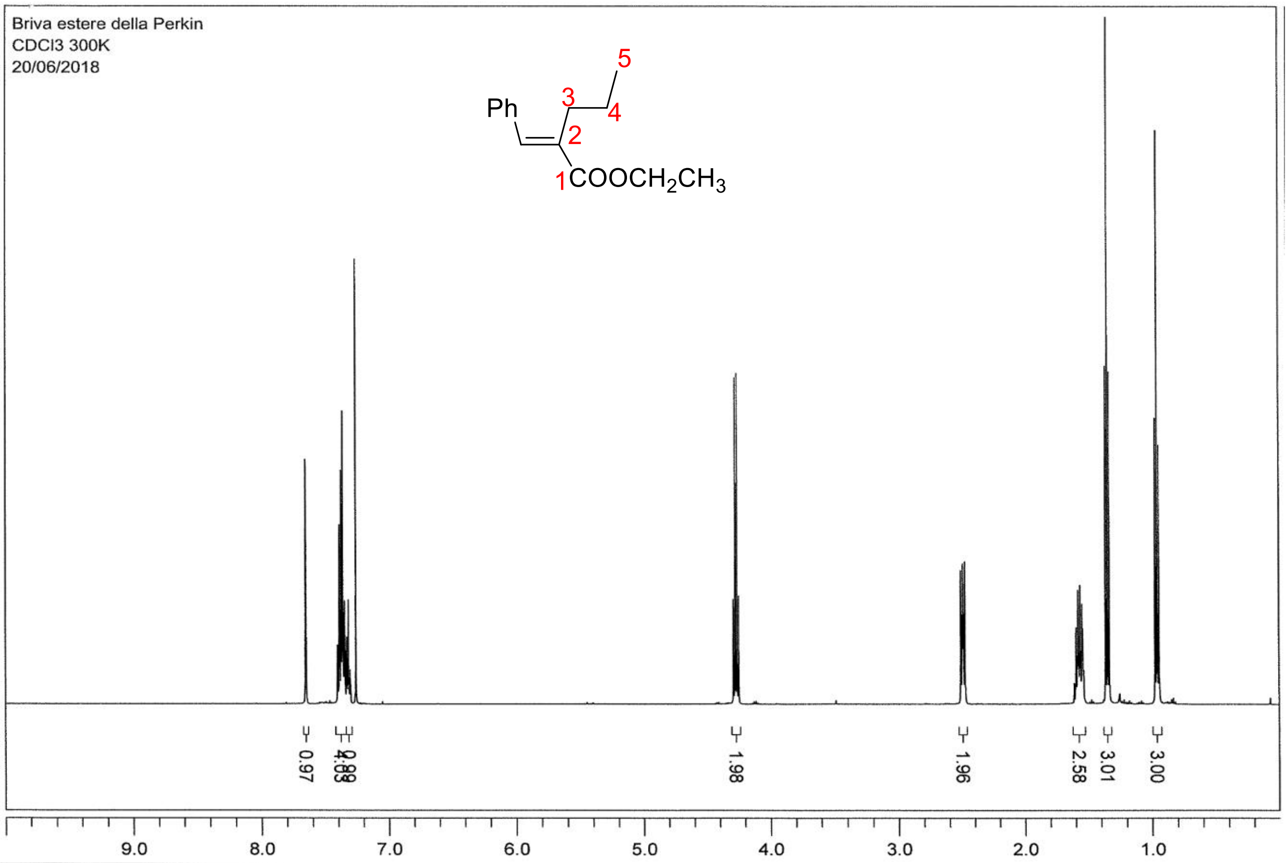

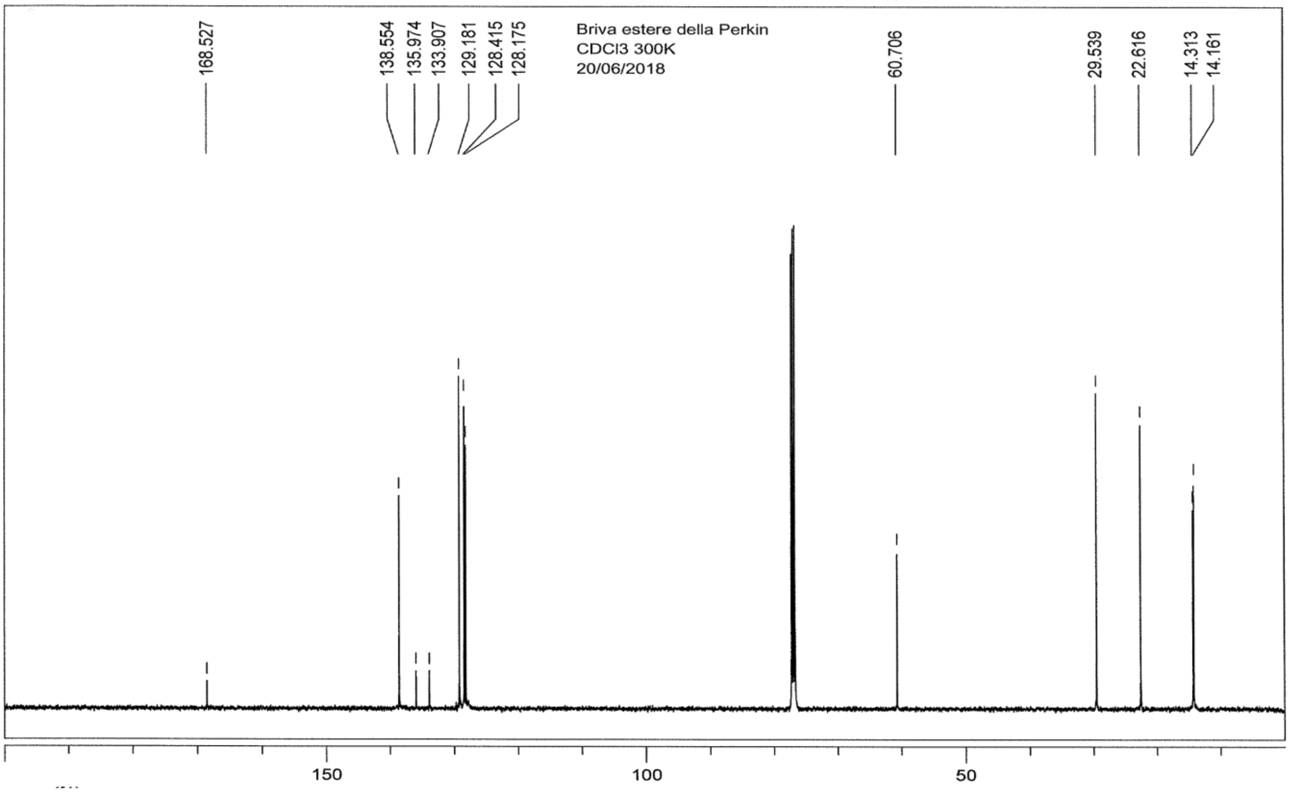

(R,S)-2-benzylpentan-1-ol **8**

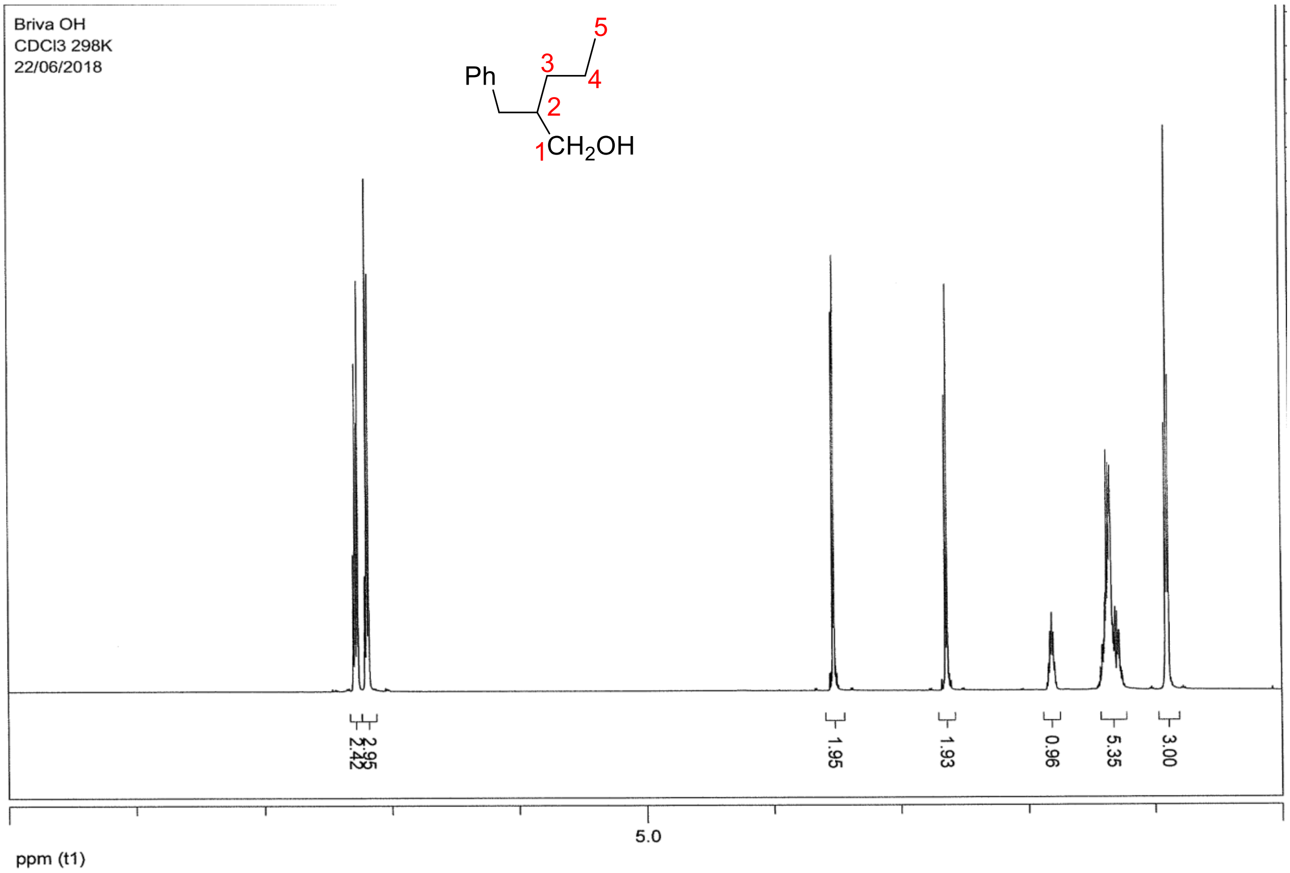

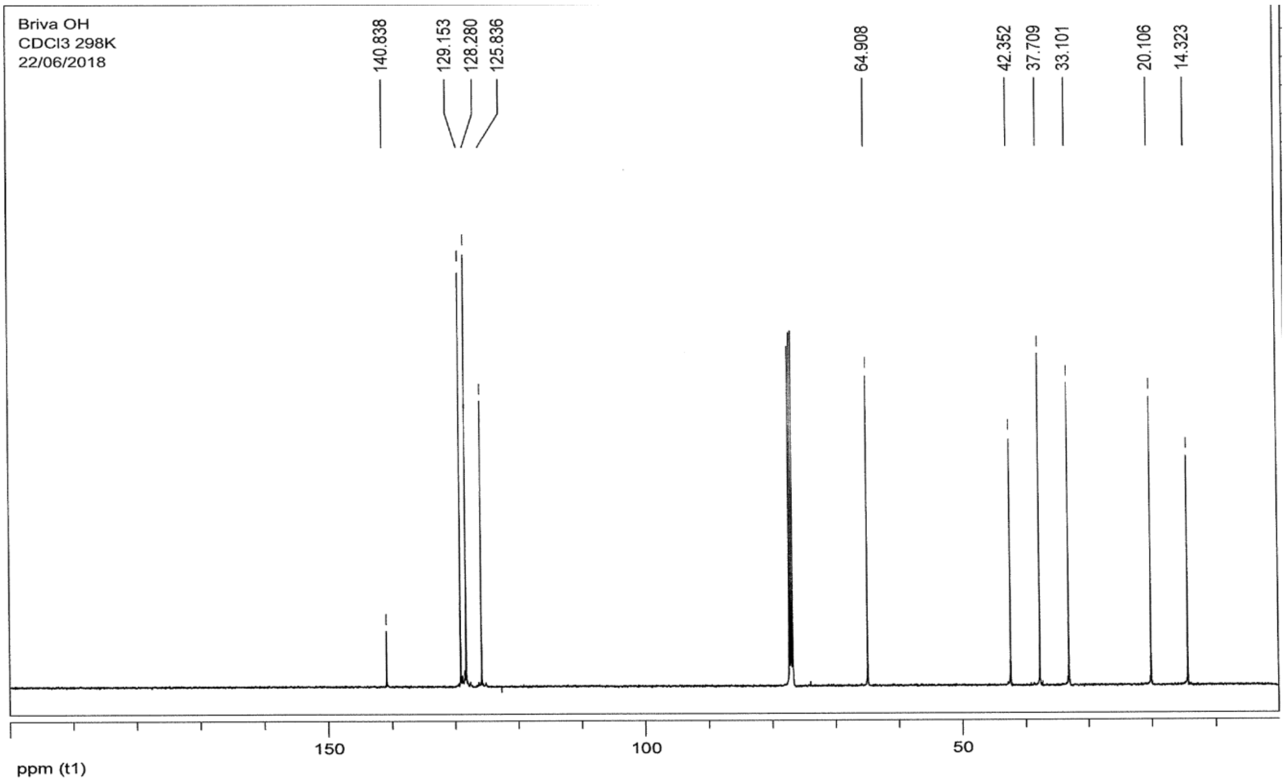

(R)-2-benzylpentan-1-ol, acetate **12**

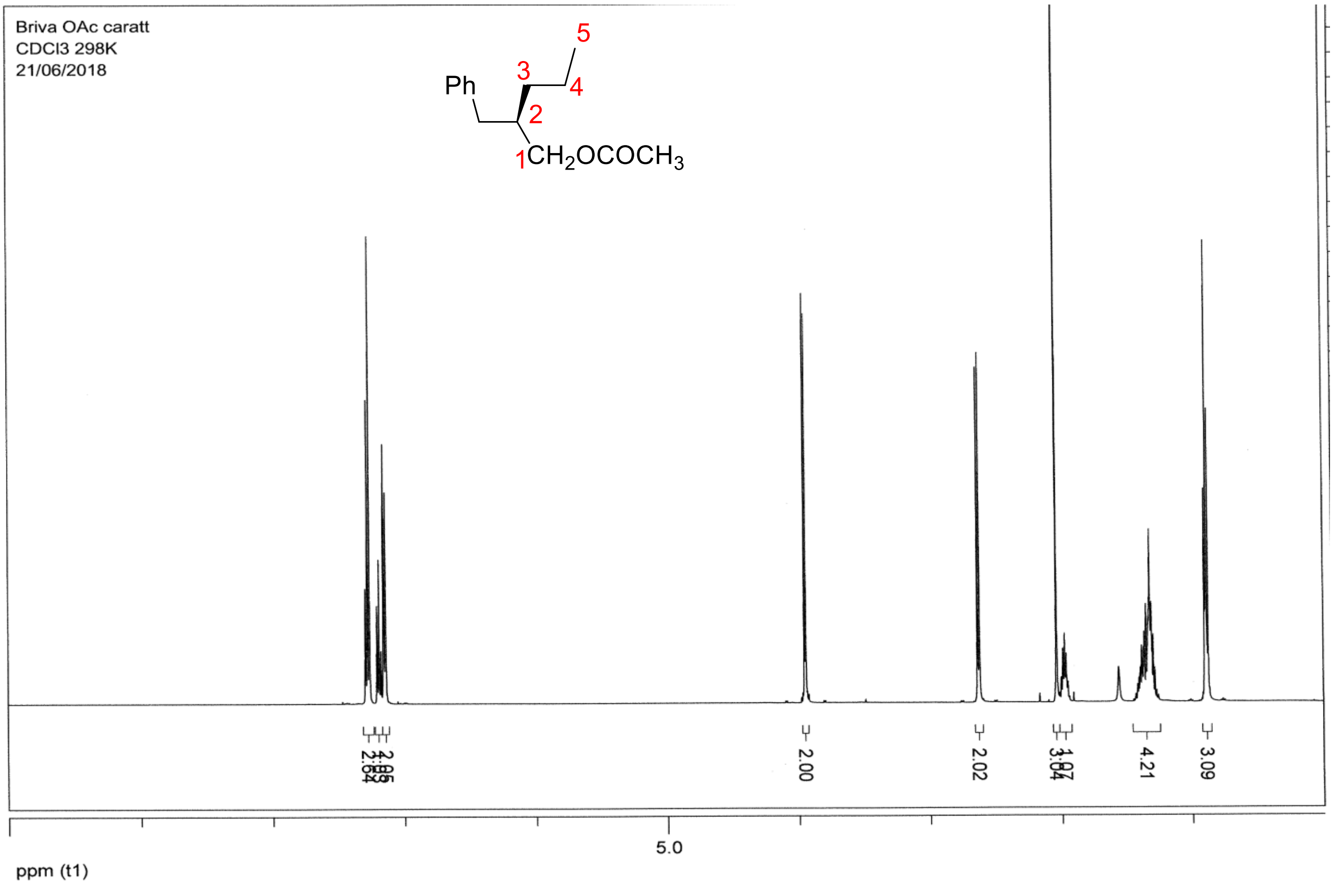

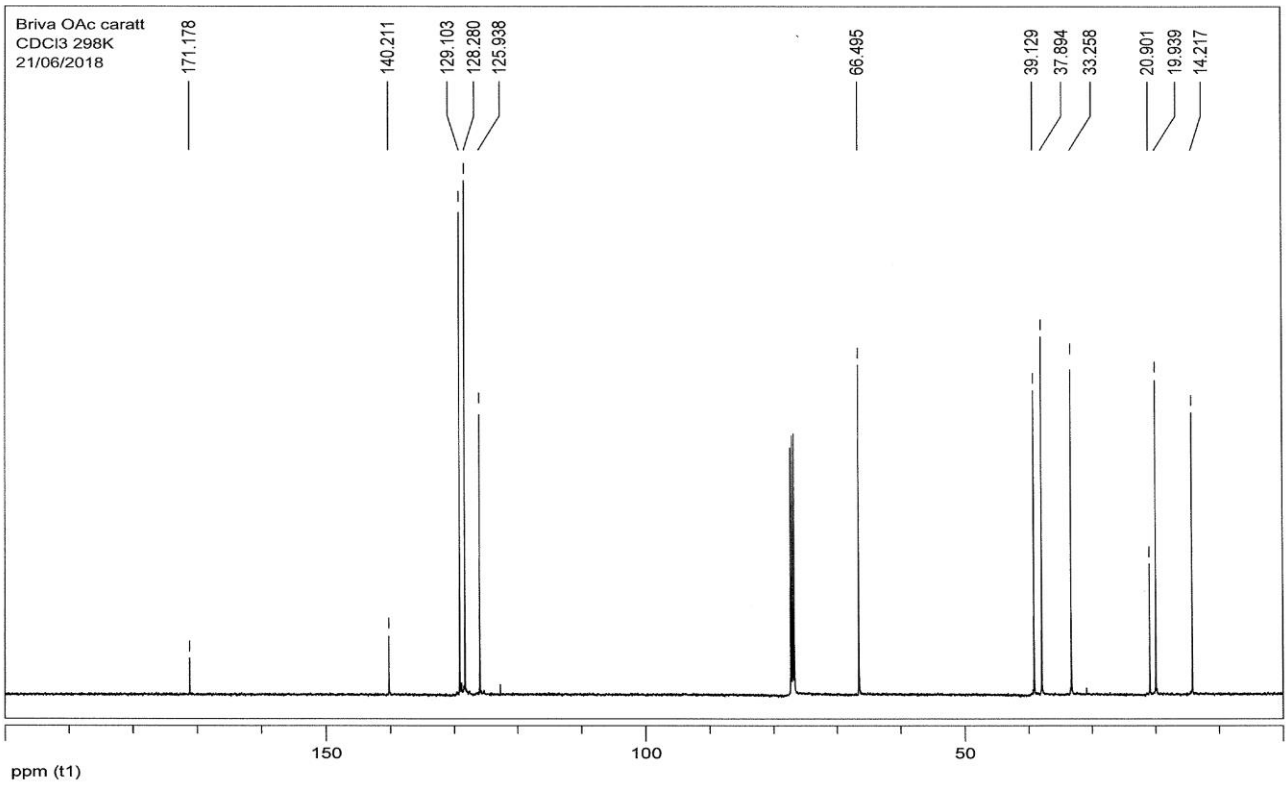

(R)-3-(acetoxymethyl)hexanoic acid **13**

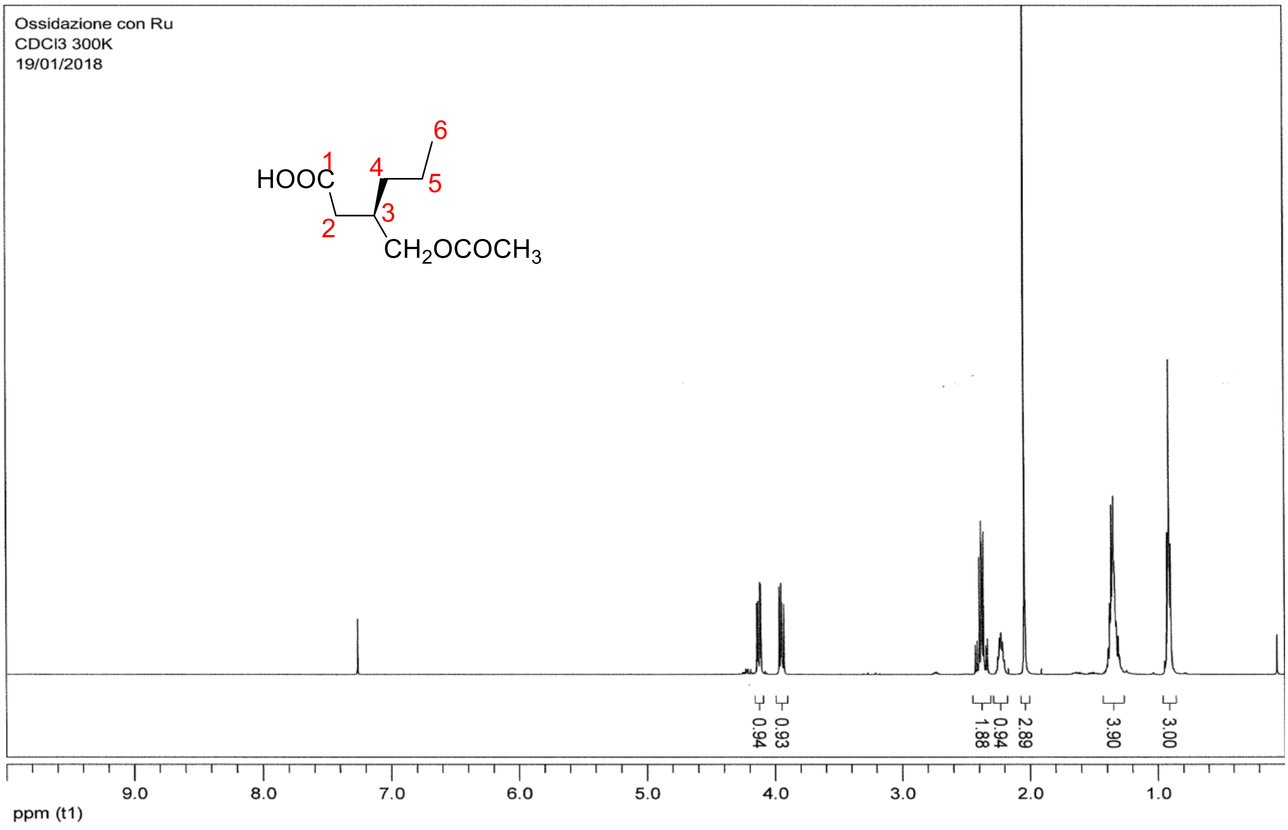

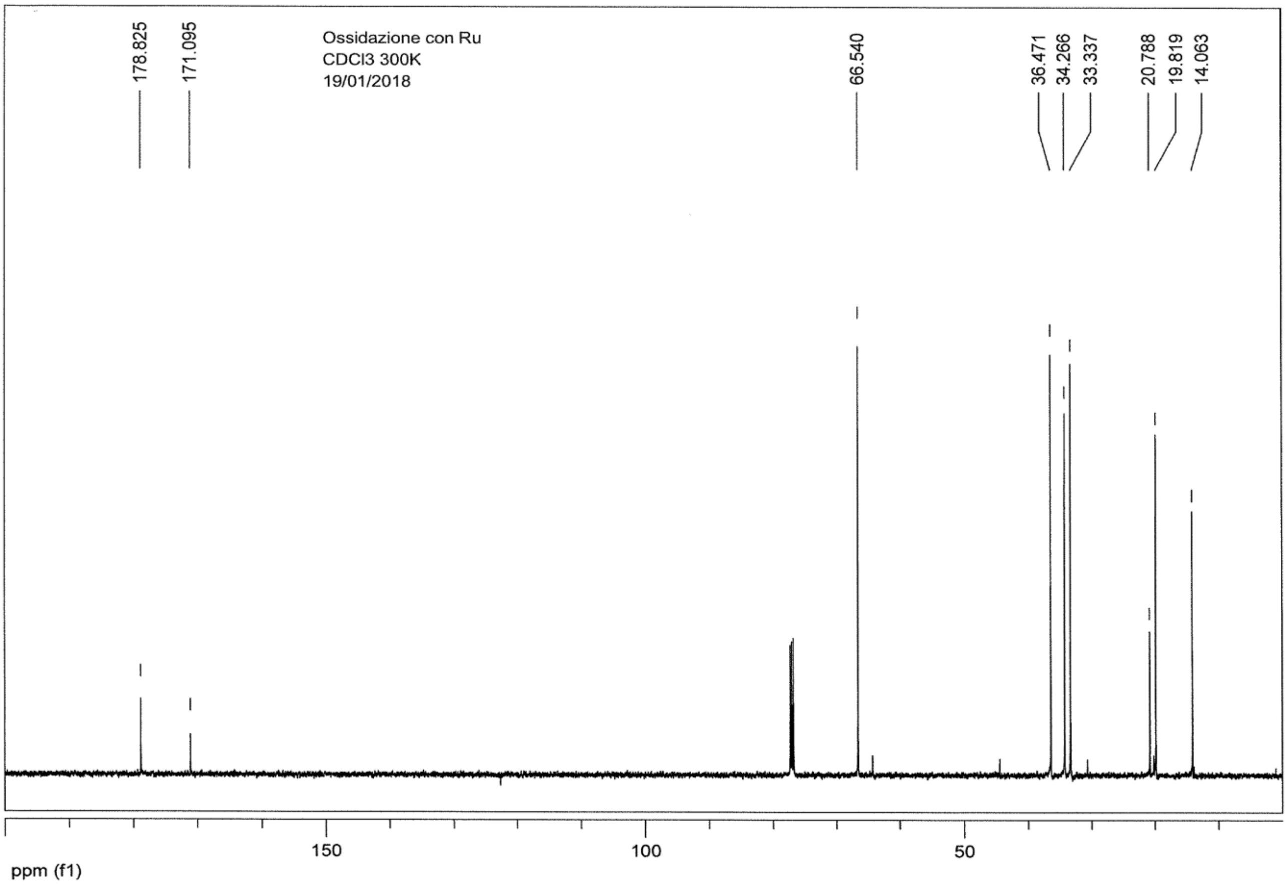

(R)-4-propyldihydrofuran-2(3H)-one **4**

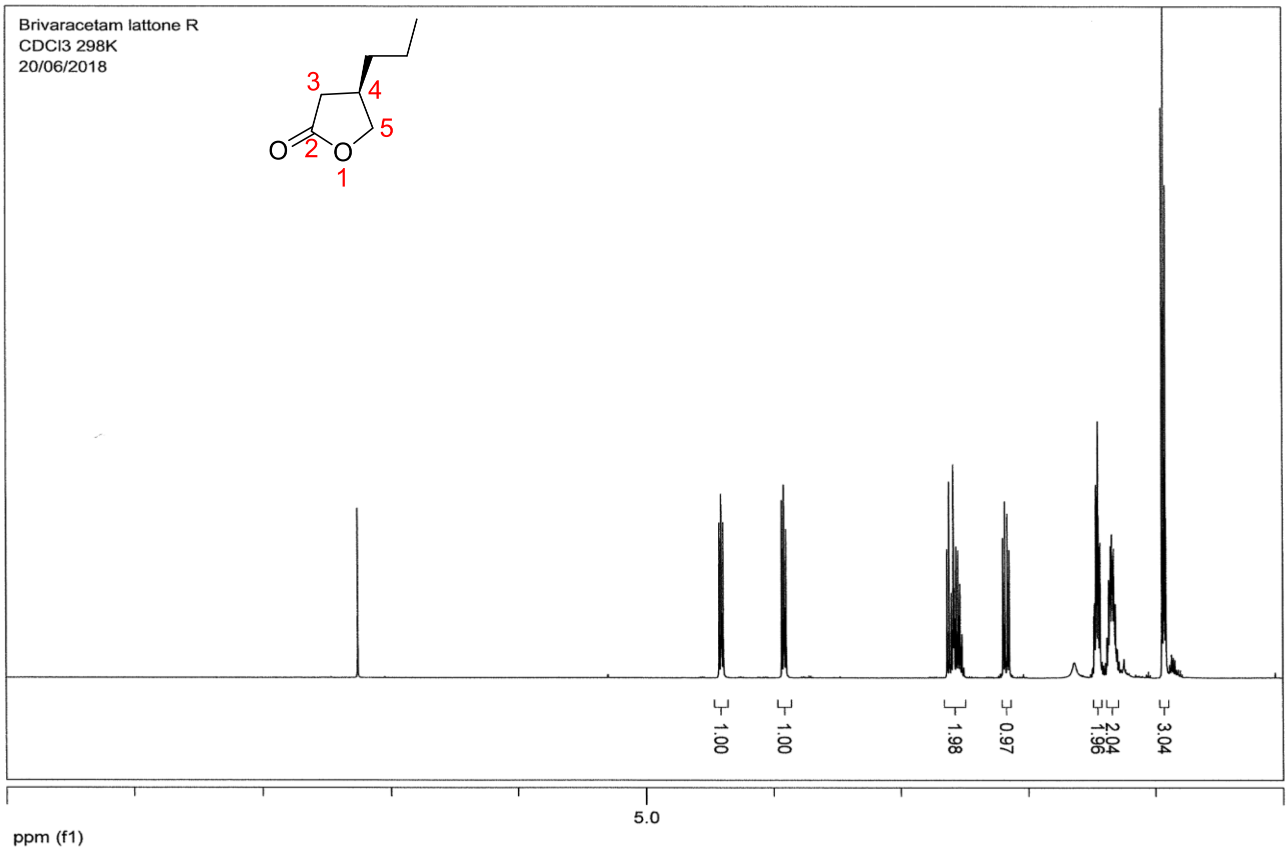

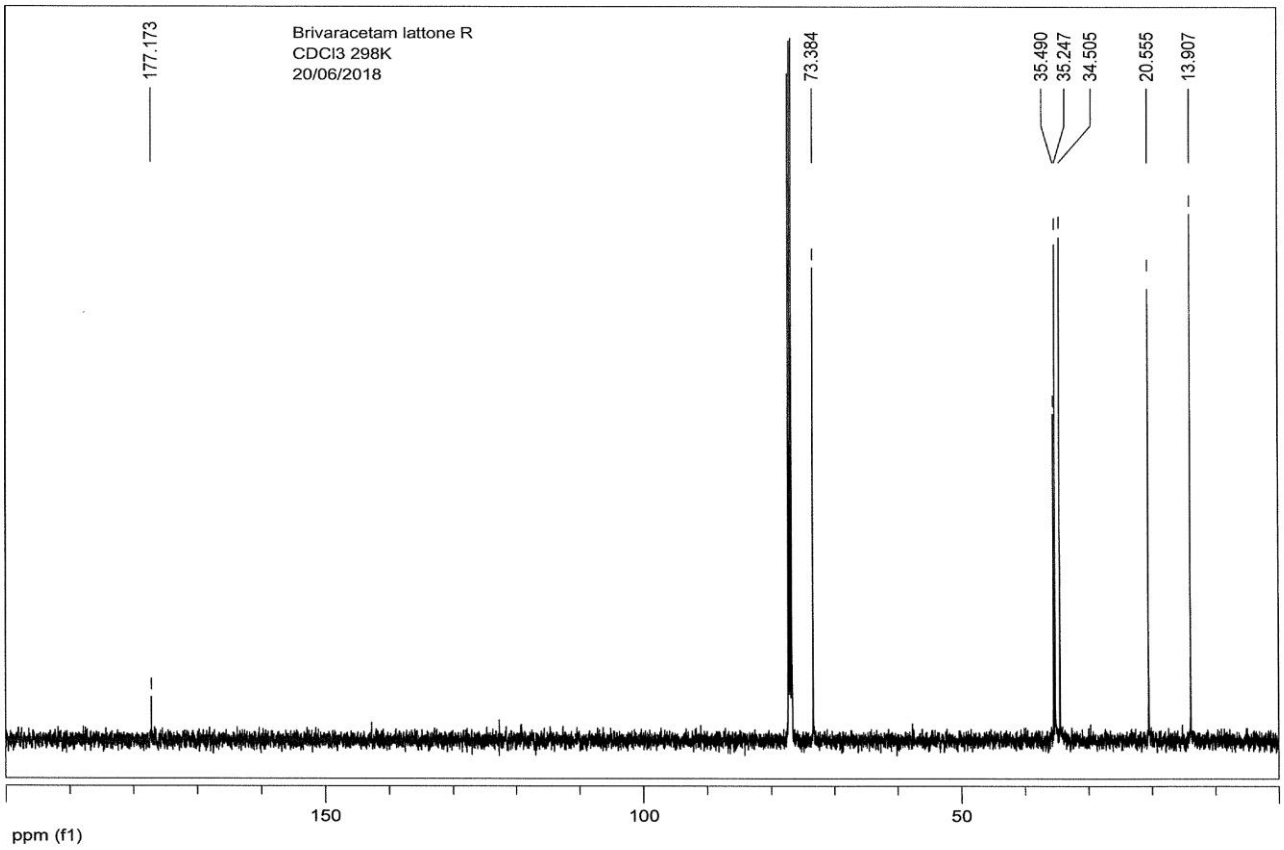

(R)-2-(2-oxo-2-(((R)-1-phenylethyl)amino)ethyl)pentyl acetate **15**

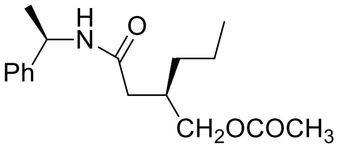

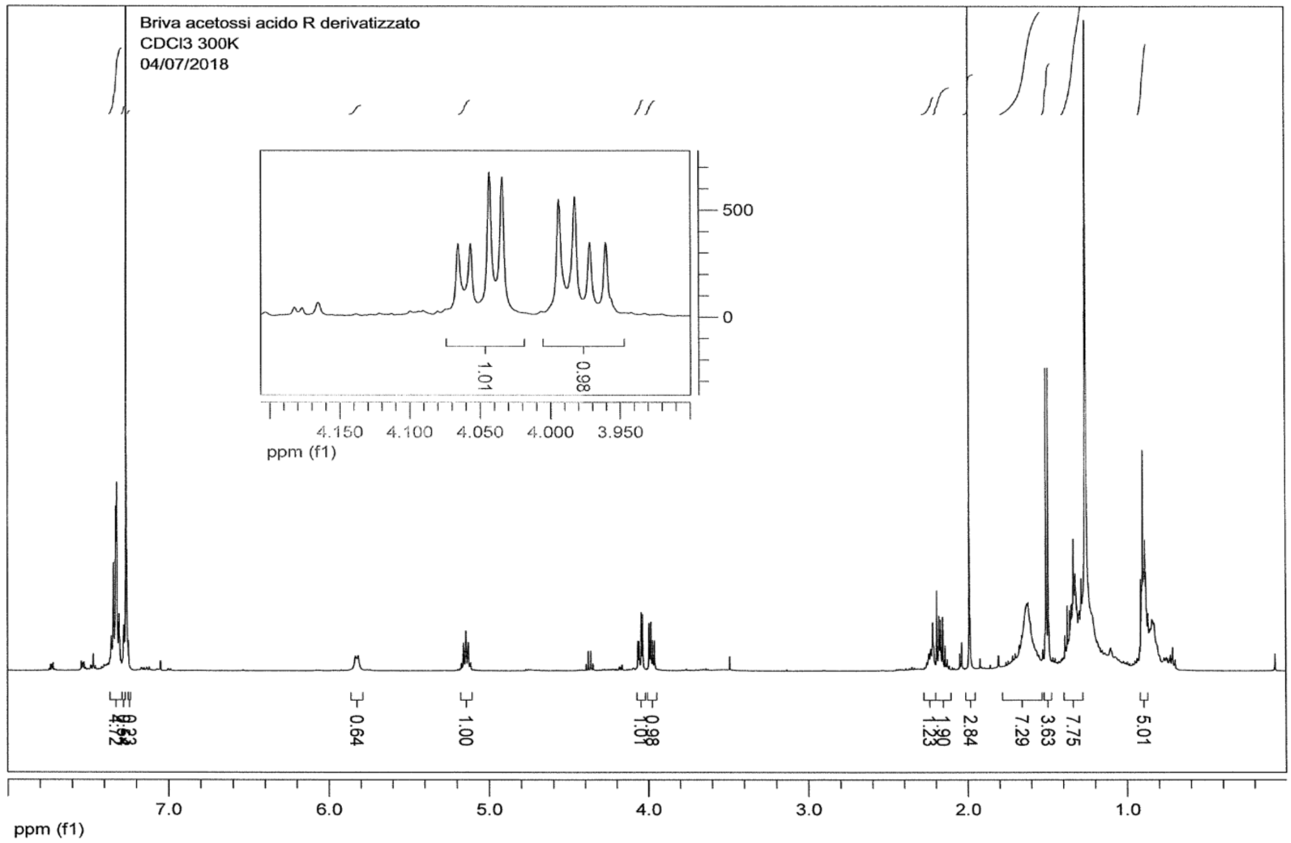

(R,S)-2-(2-oxo-2-(((R)-1-phenylethyl)amino)ethyl)pentyl acetate **15**

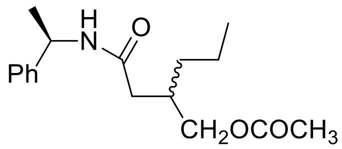

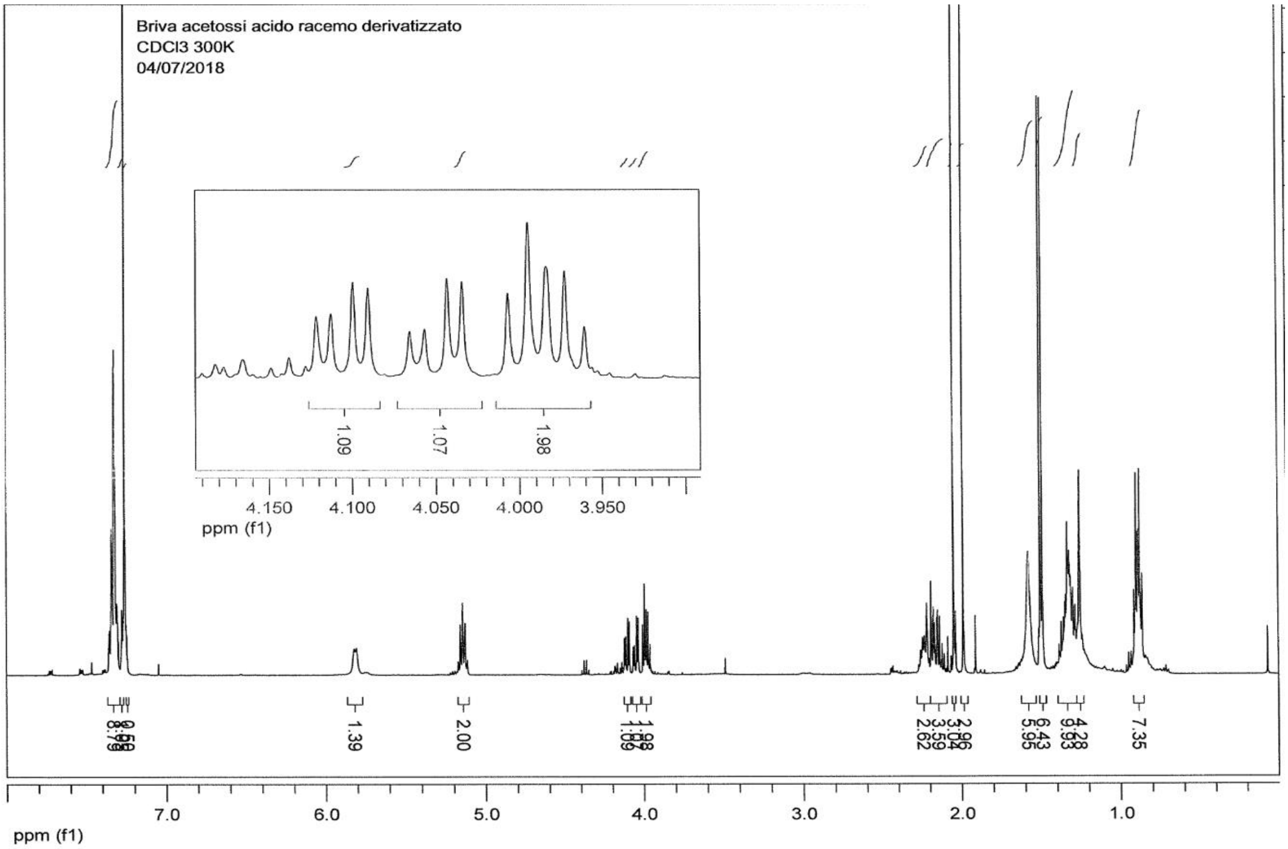

IR spectra

(E)-Ethyl-2-benzylidenepentanoate **11**

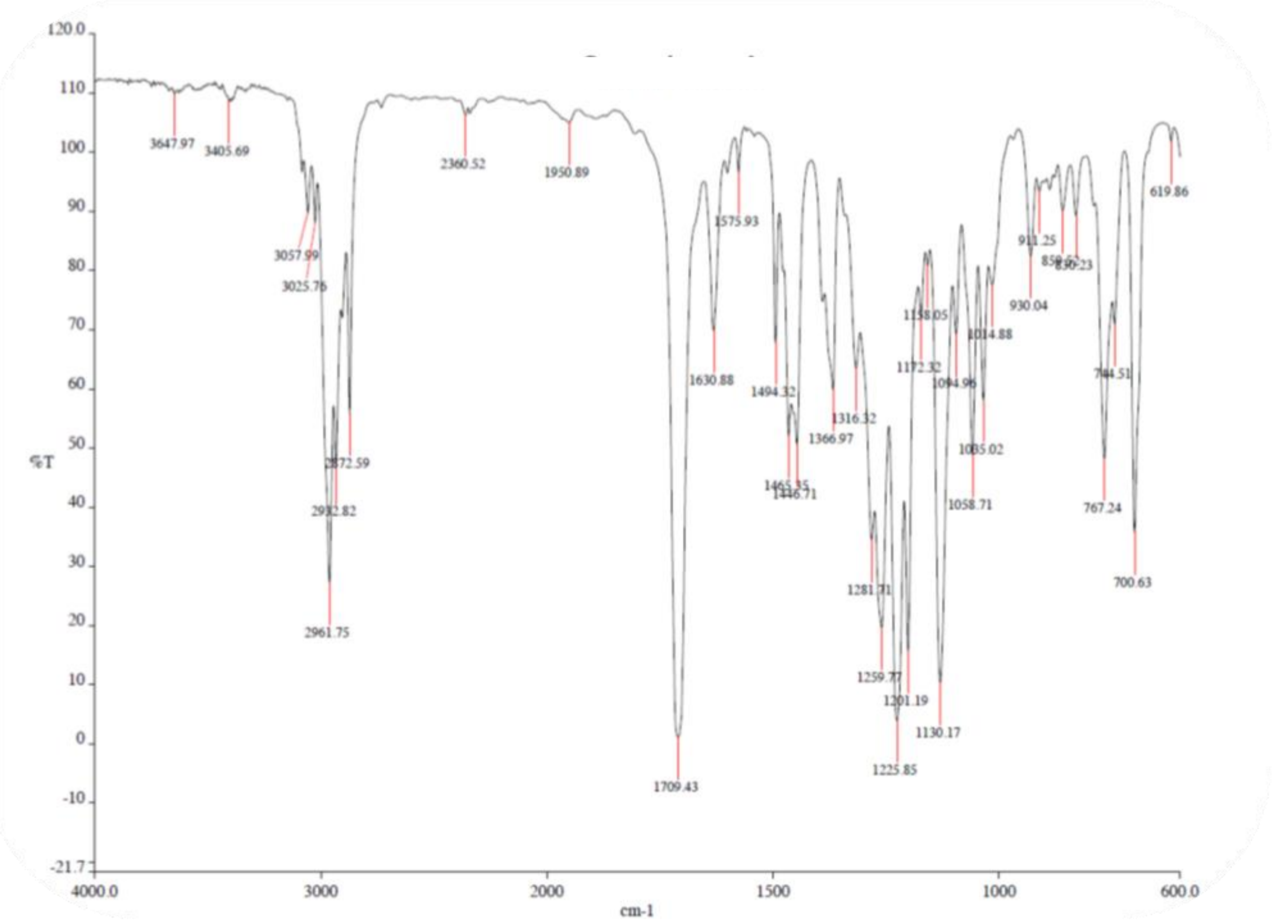

(R,S)-2-benzylpentan-1-ol **8**

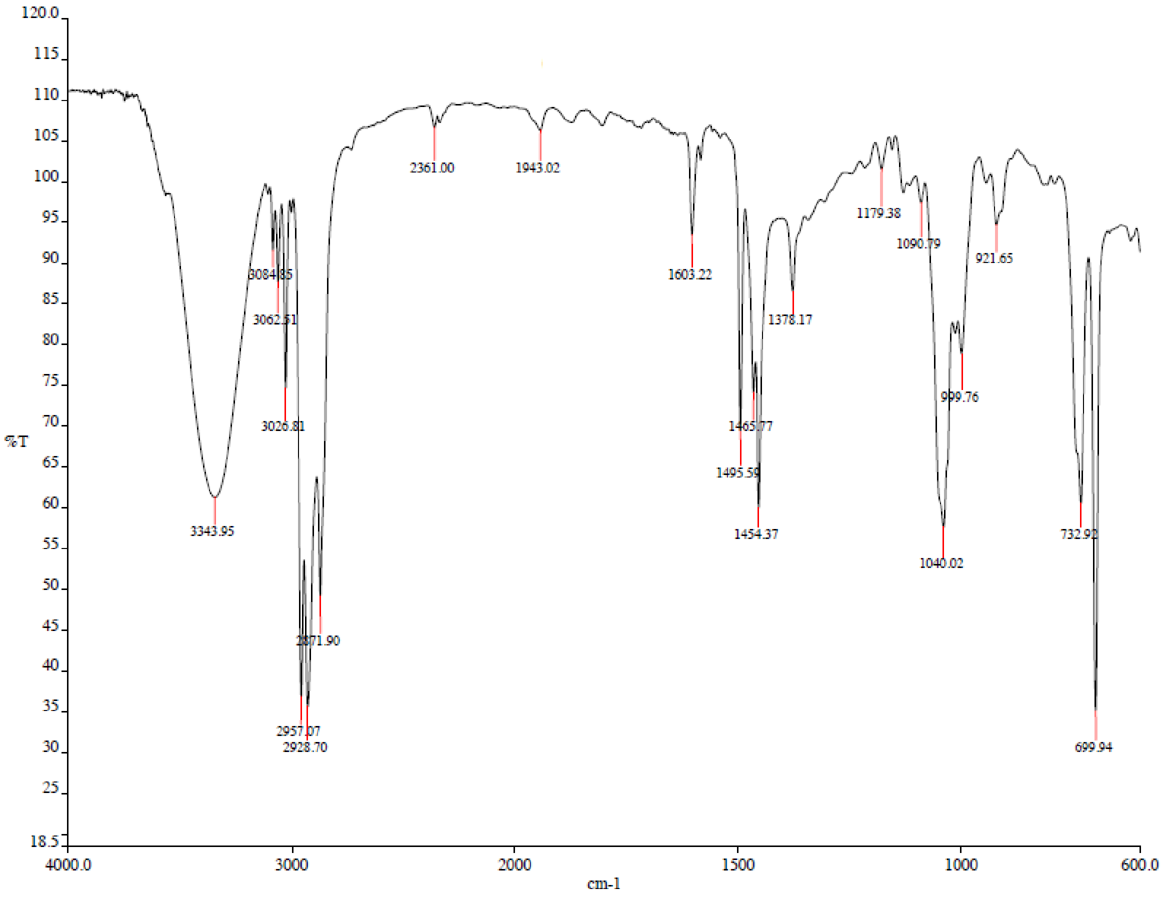

(R)-2-benzylpentan-1-ol, acetate **12**

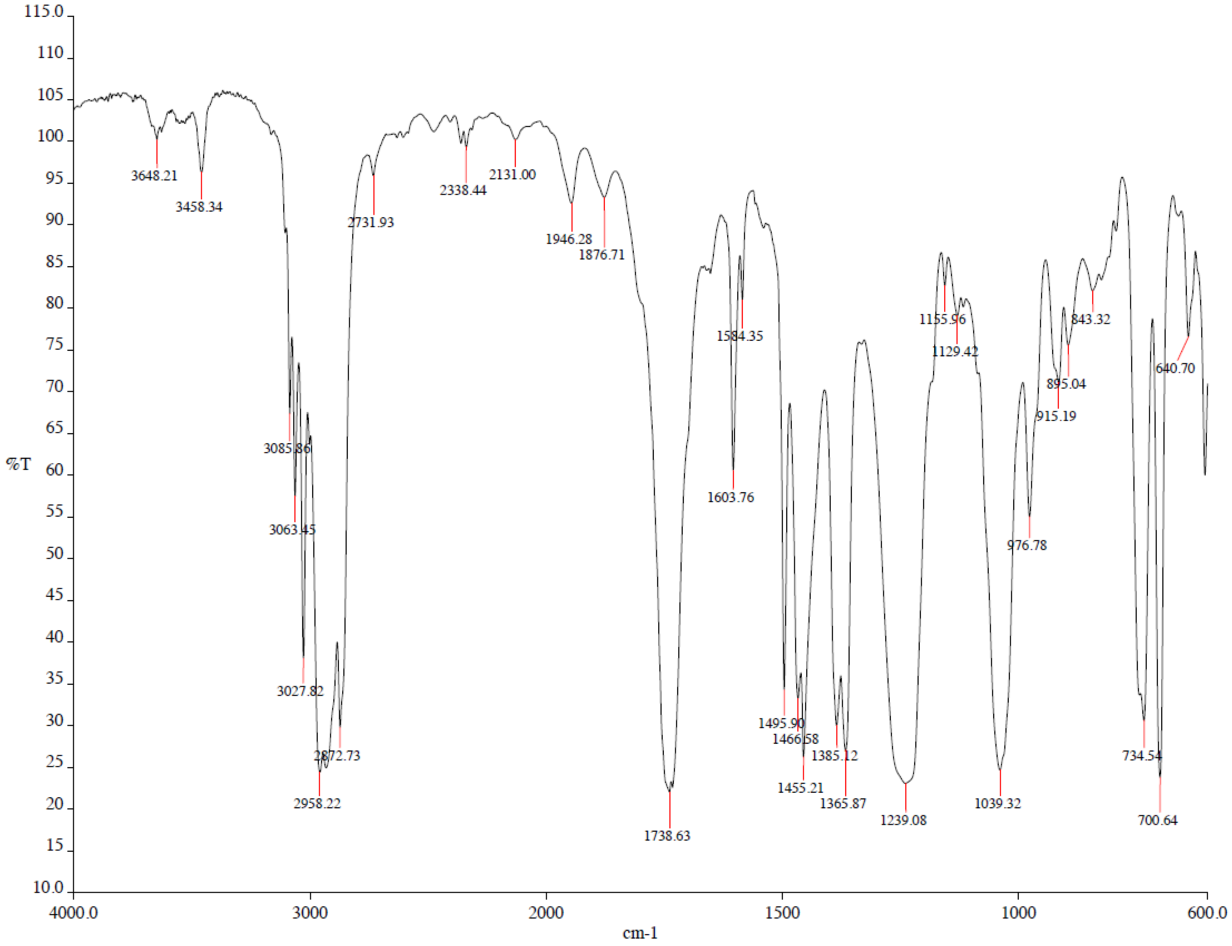

(R)-3-(acetoxymethyl)hexanoic acid **13**

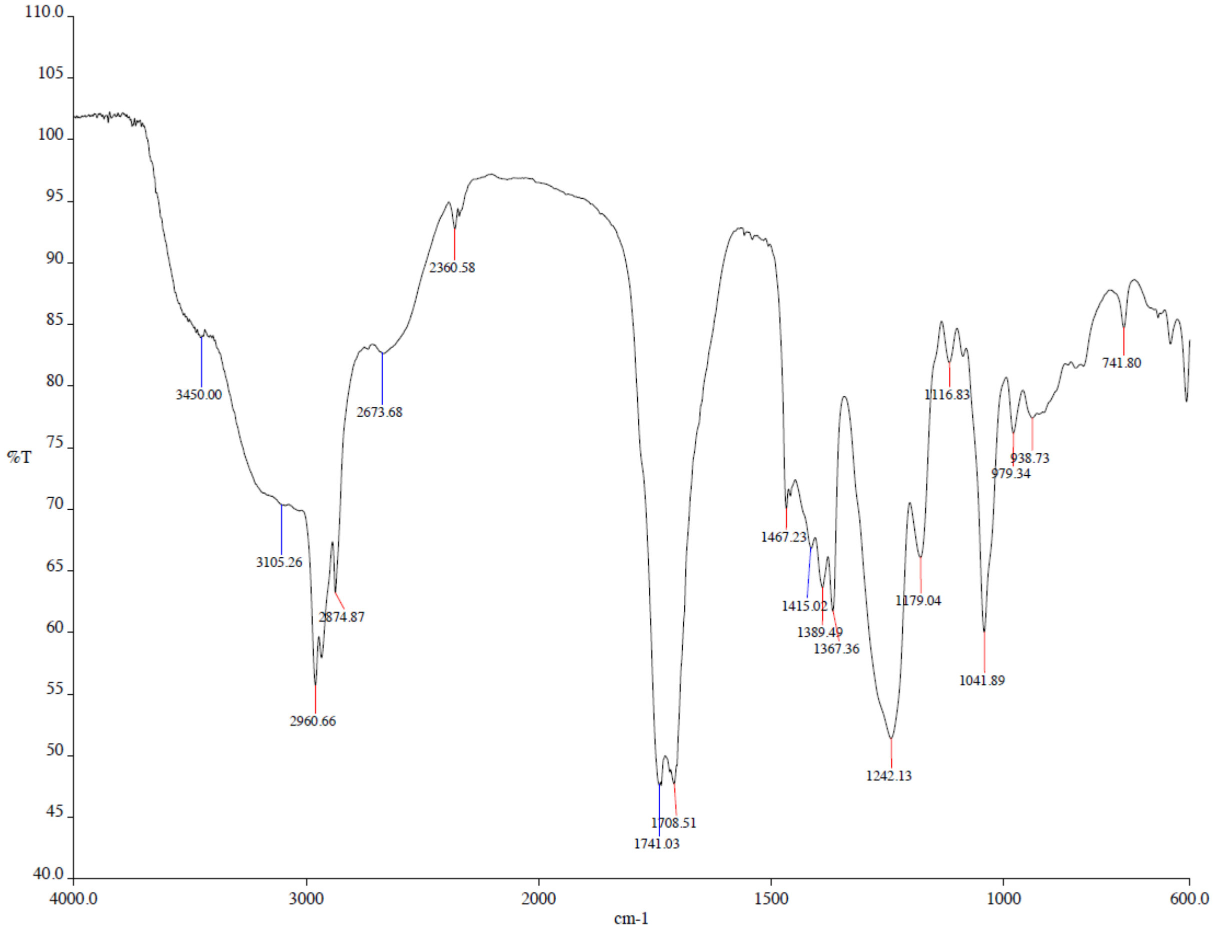

(R)-4-propyldihydrofuran-2(3H)-one **4**

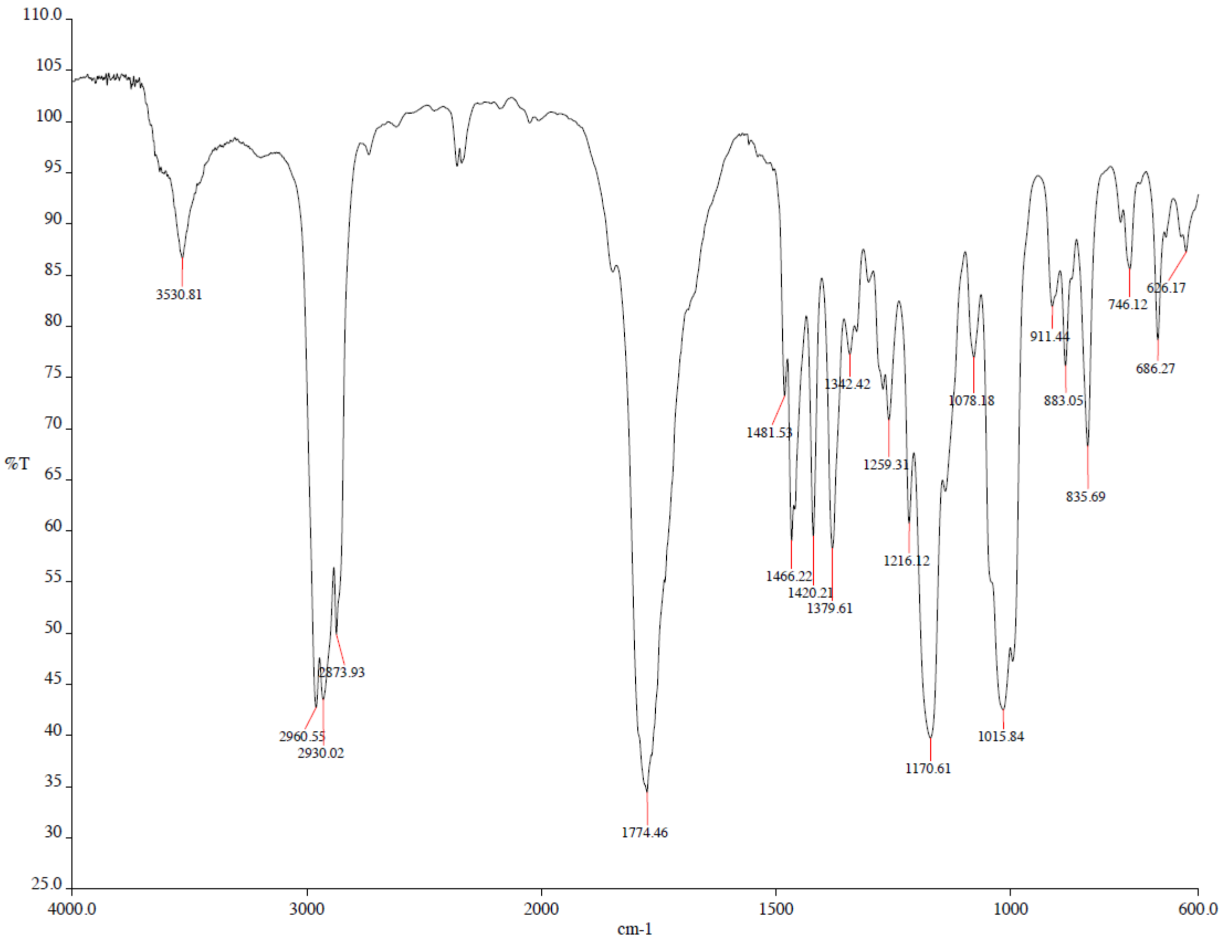

MS spectra

(E)-Ethyl-2-benzylidenepentanoate **11**

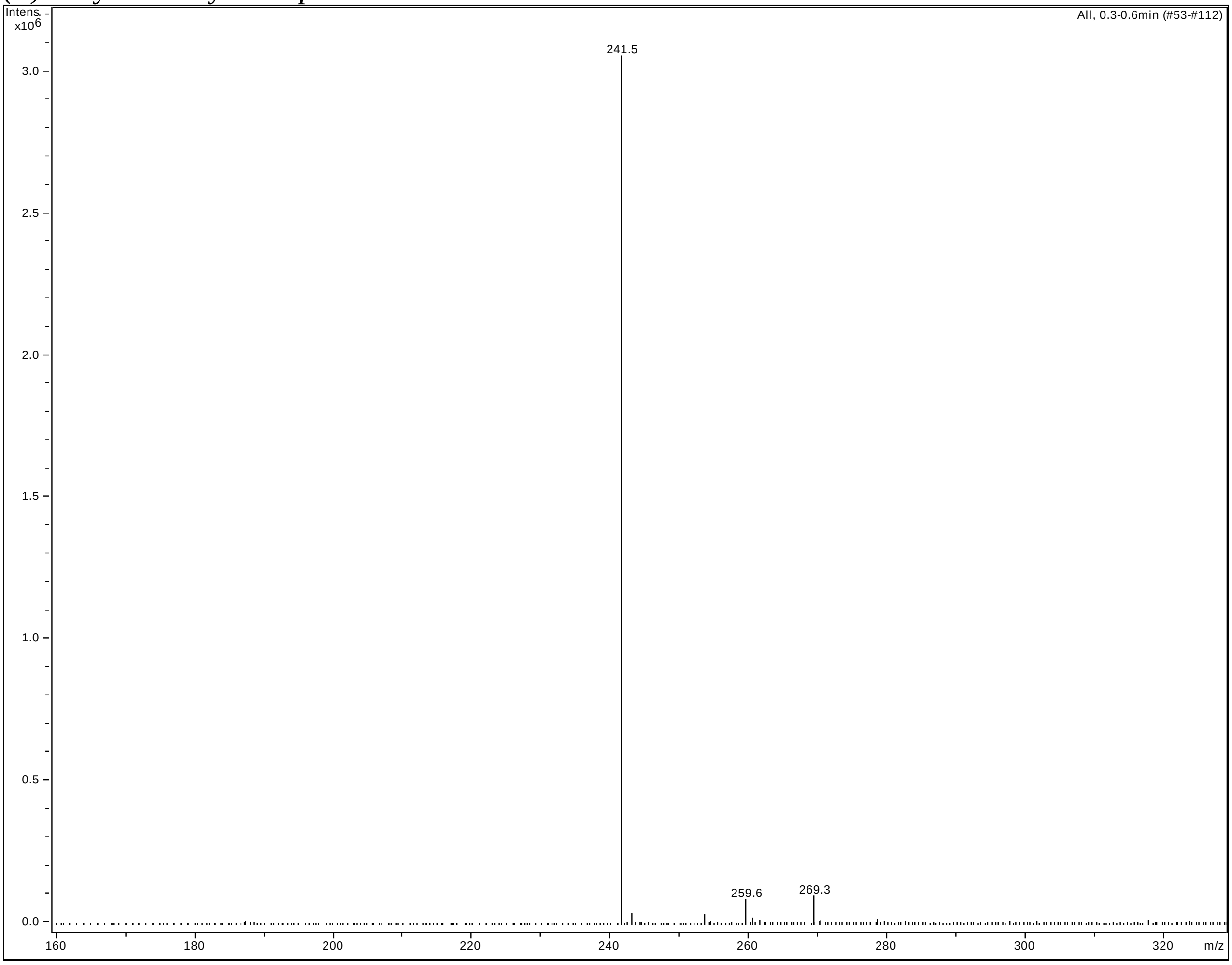

(R,S)-2-benzylpentan-1-ol **8**

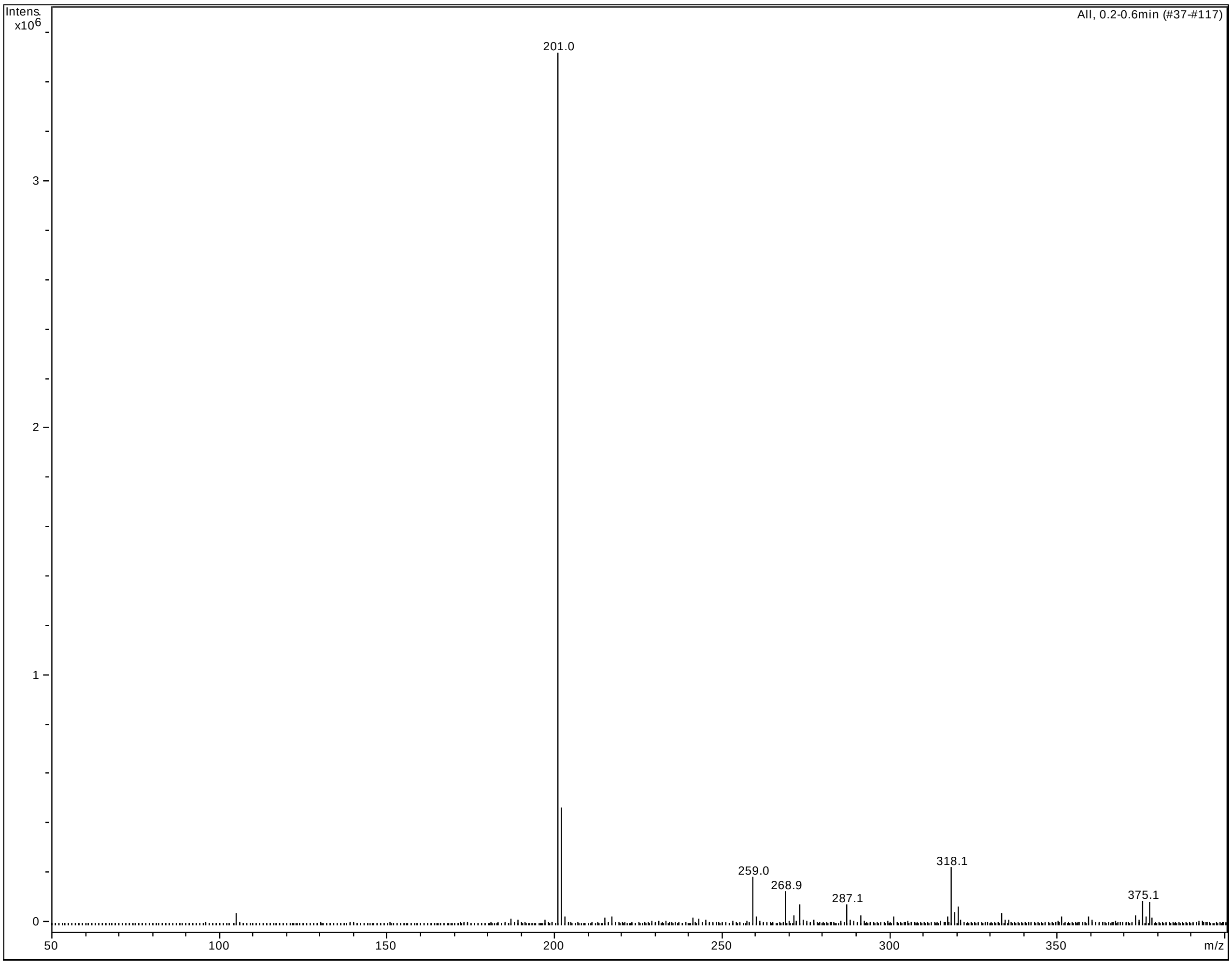

(R)-2-benzylpentan-1-ol, acetate **12**

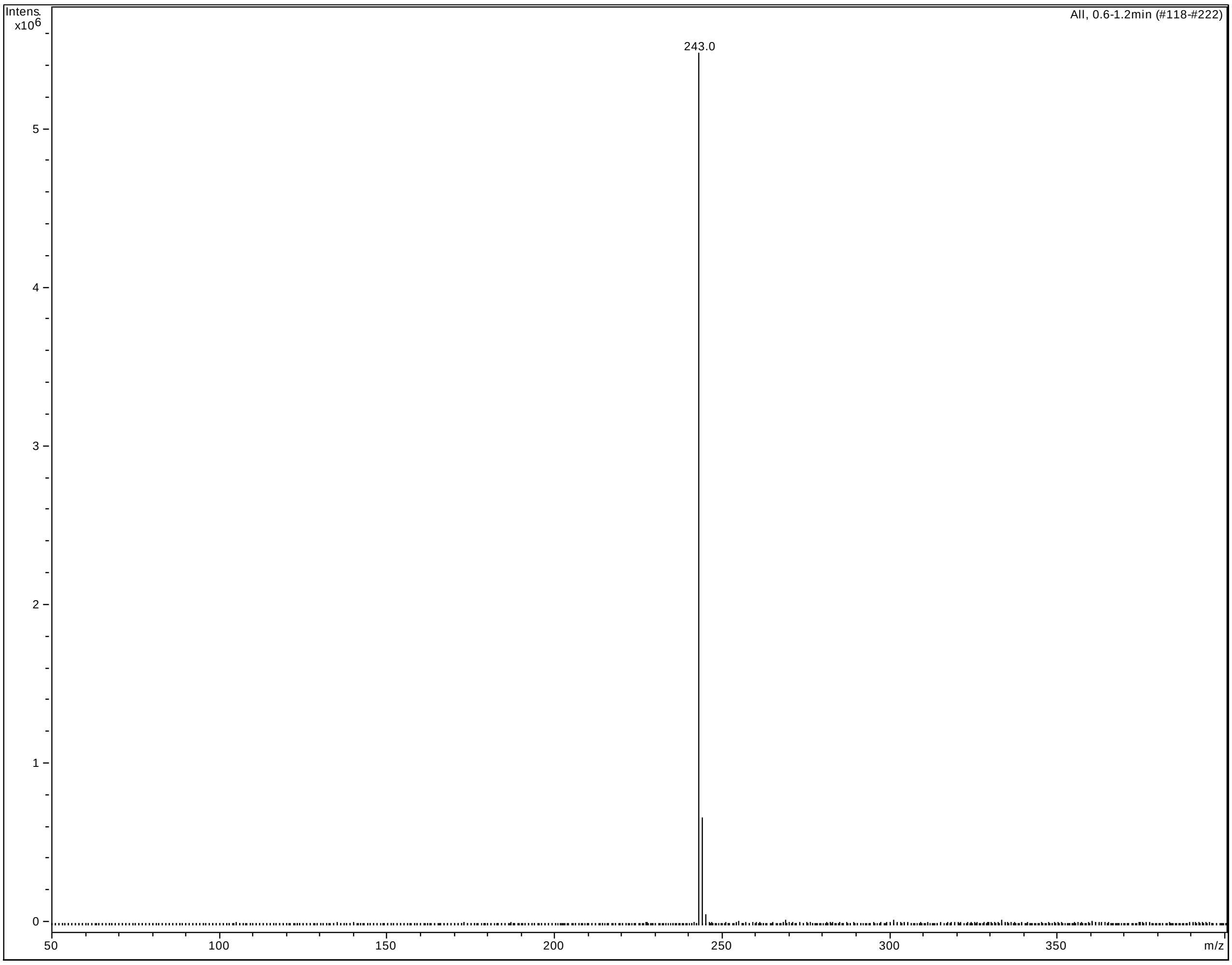

(R)-3-(acetoxymethyl)hexanoic acid **13**

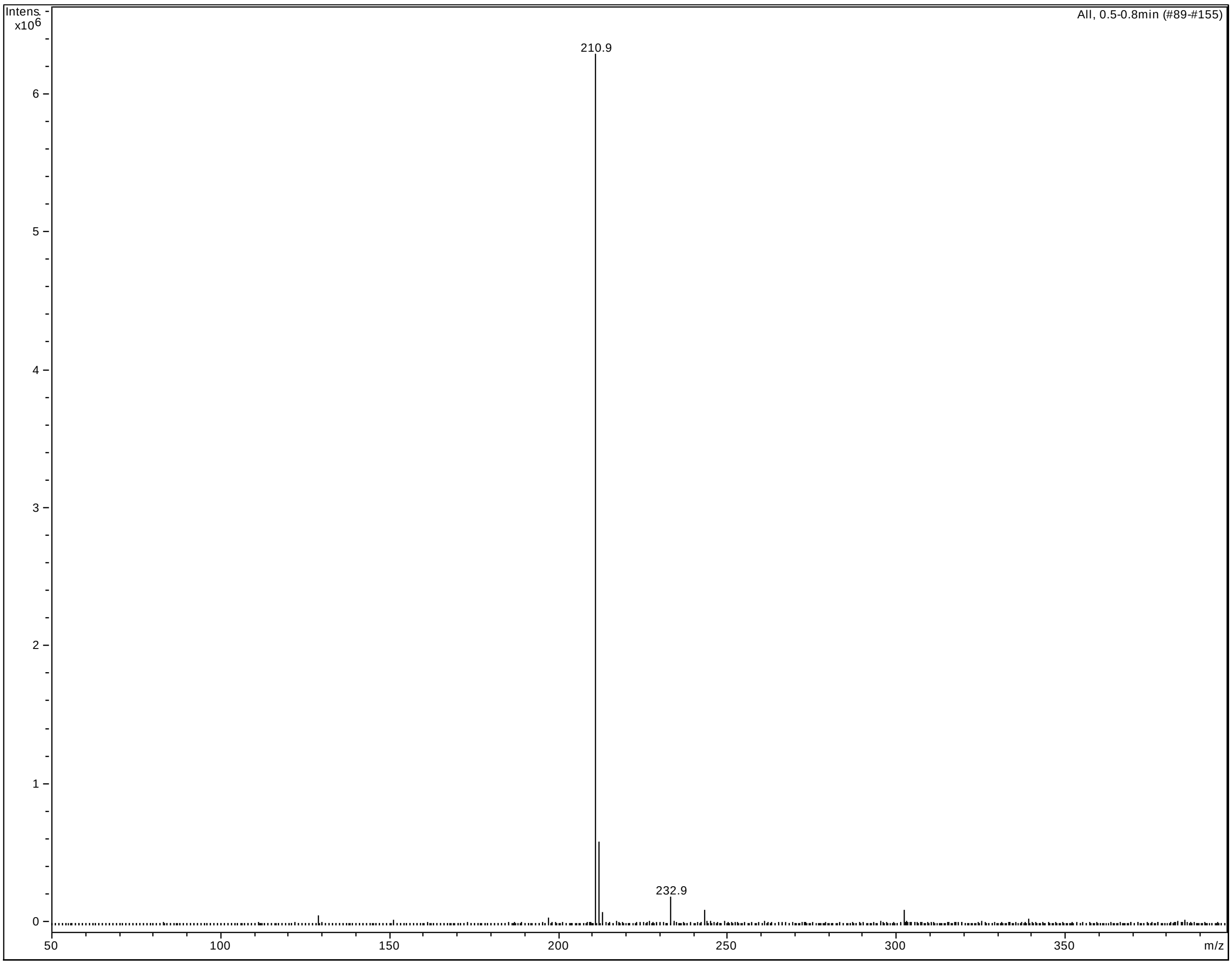

(R)-4-propyldihydrofuran-2(3H)-one **4**

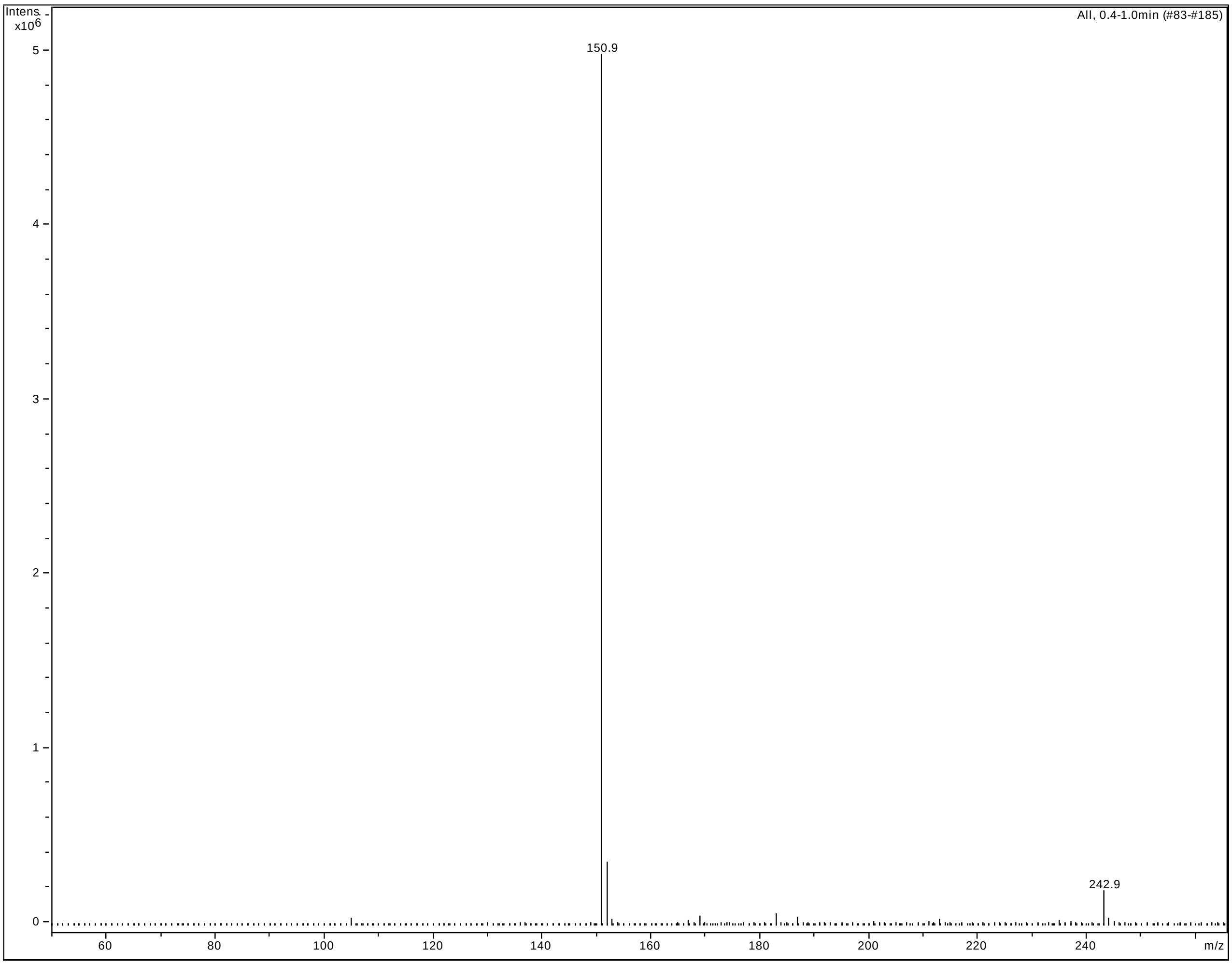

HRMS spectra

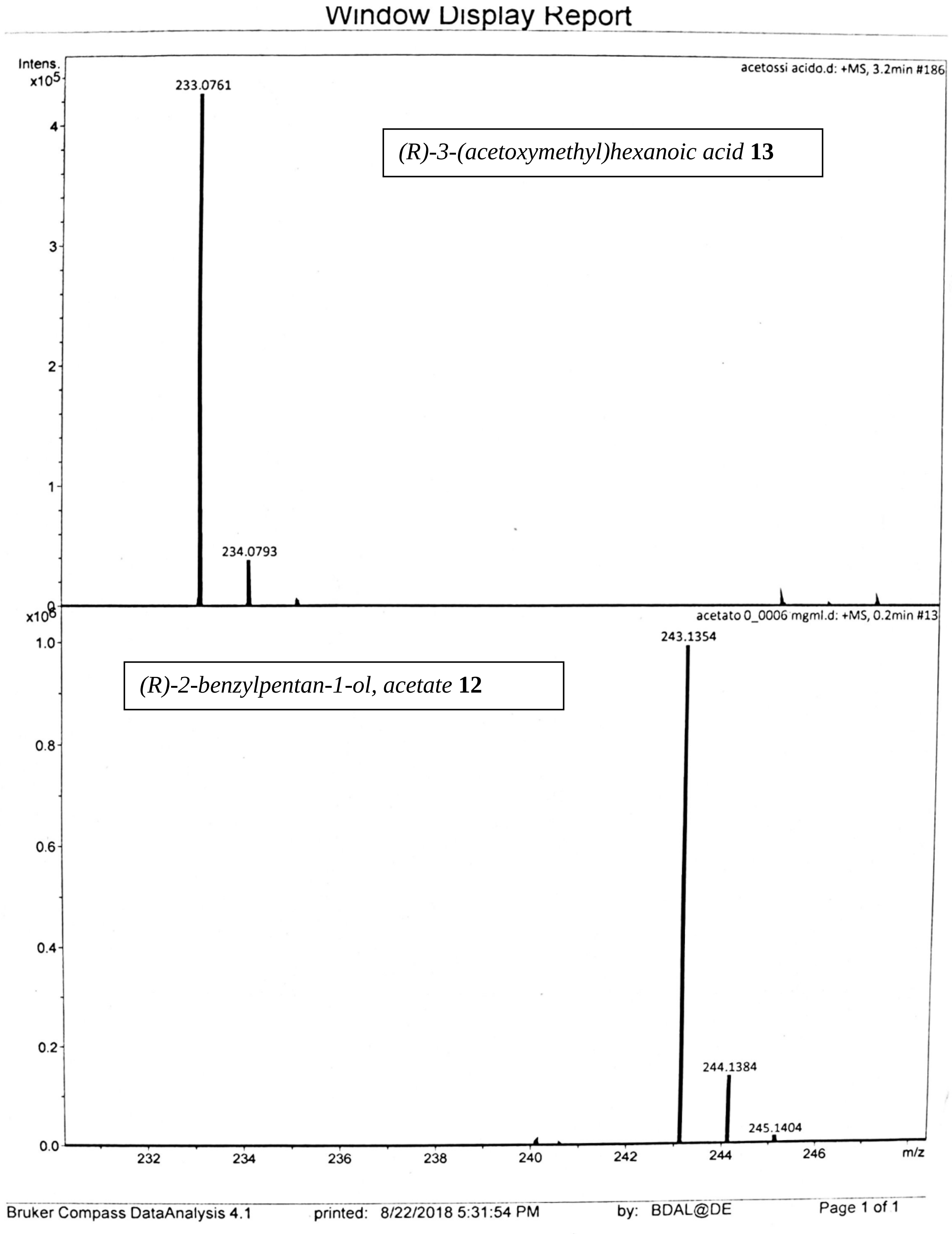

Chromatograms

HPLC analysis of crude obtained by PFL-catalyzed irreversible transesterification

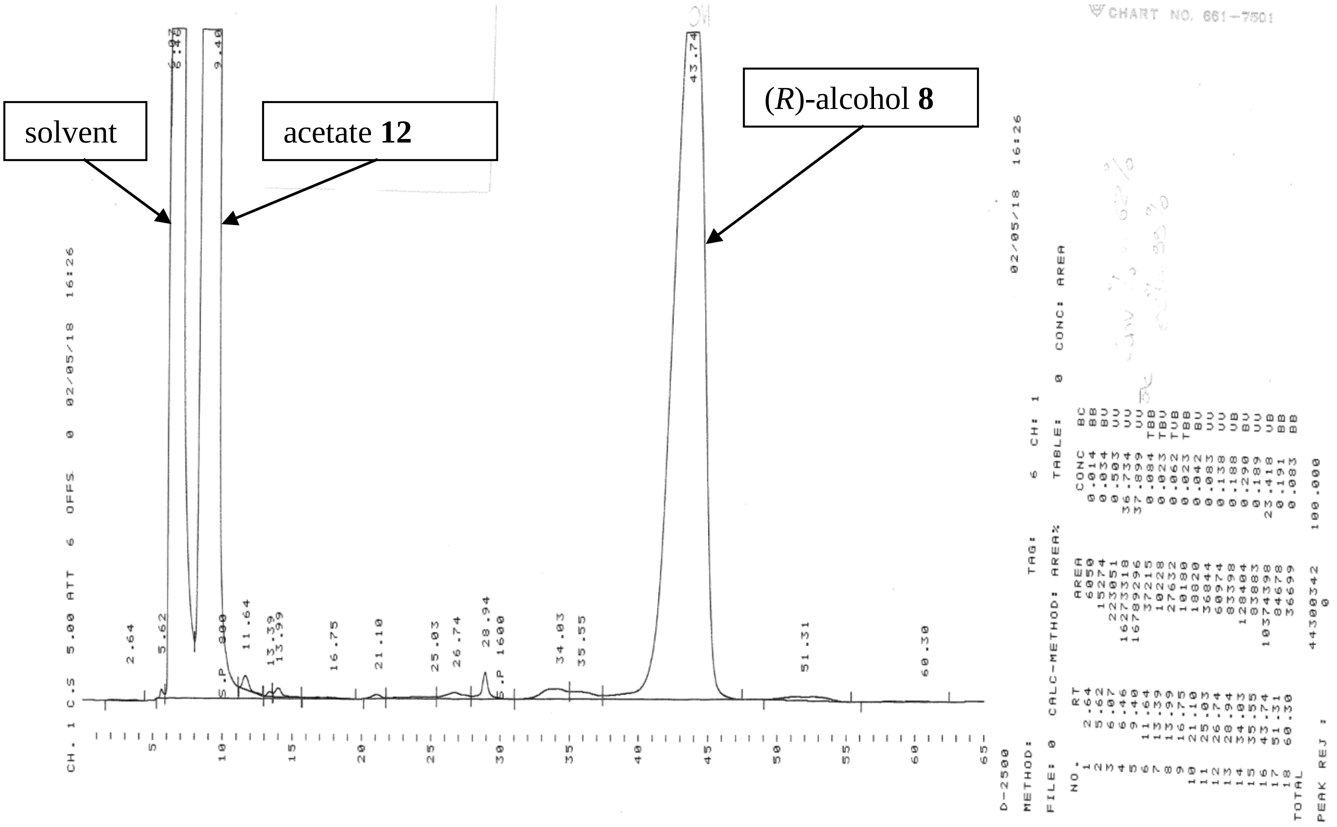

HPLC analysis of (R,S)-alcohol **8**

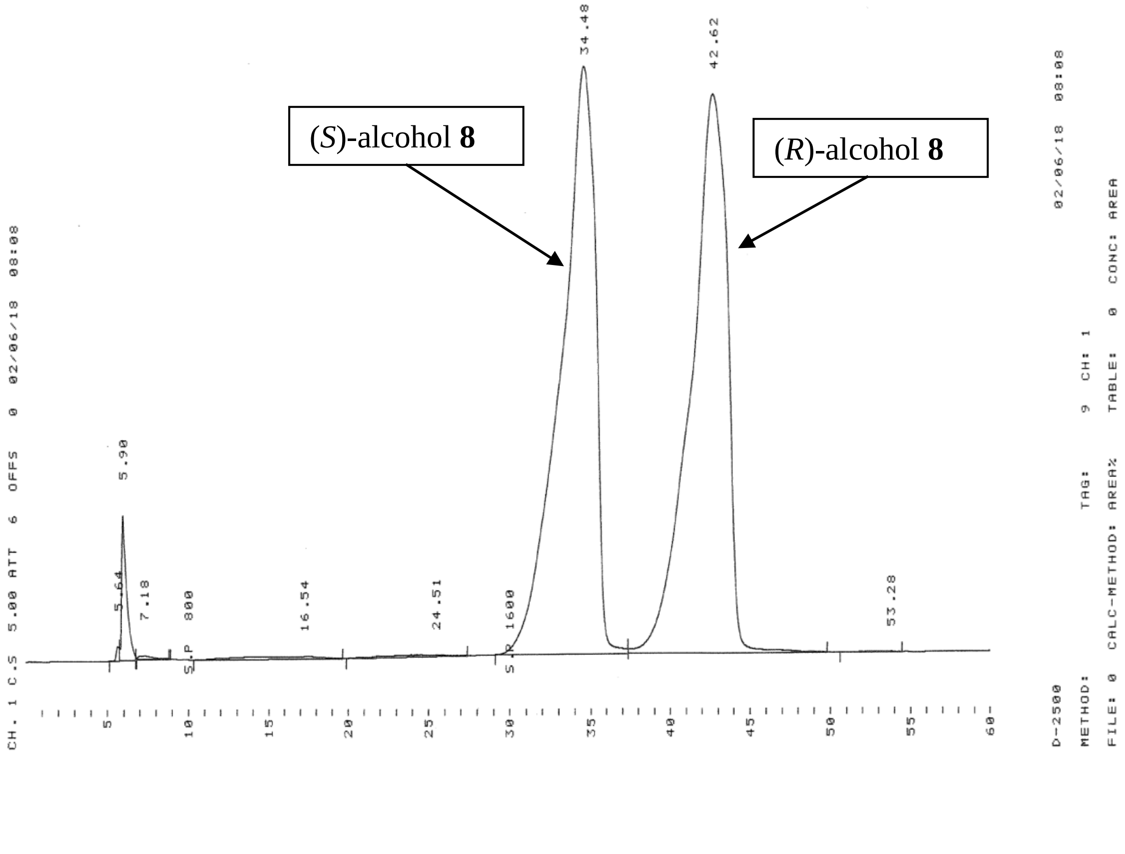

GC analysis

GC chromatogram of *(R)-4-propyldihydrofuran-2(3H)-one* **4**

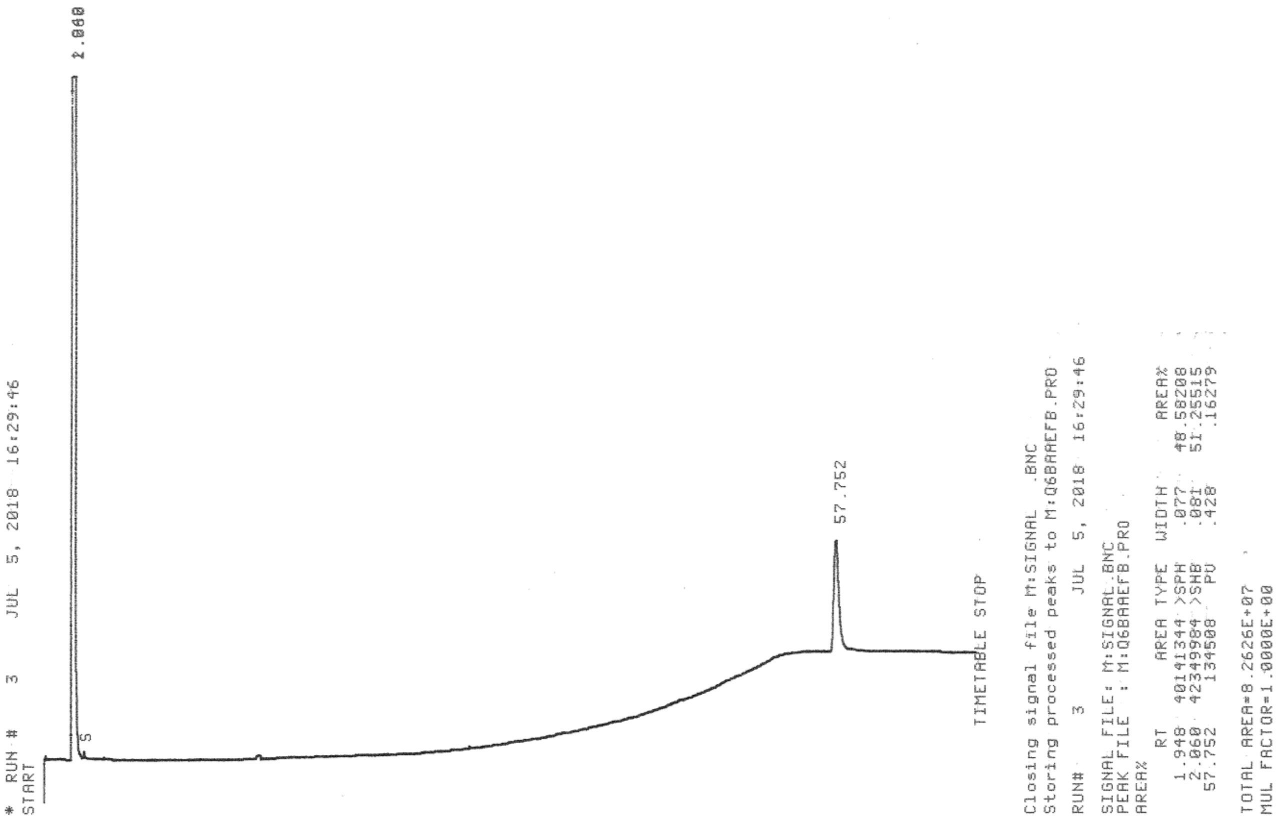

GC chromatogram of *(R,S)-4-propyldihydrofuran-2(3H)-one* **4**

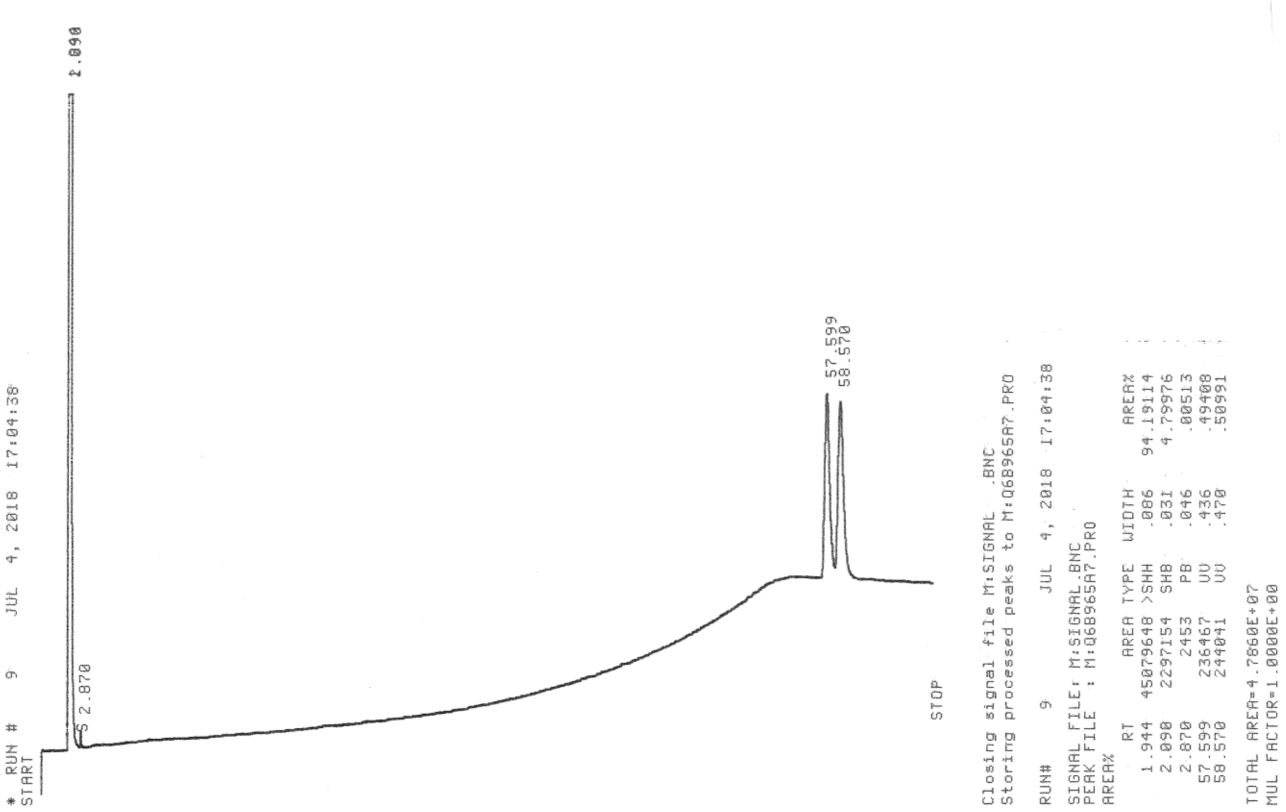

Supplement: Supplementary file 1 [file molecules-23-02206-s001.pdf]
